# Food texture influences on satiety: systematic review and meta-analysis

**DOI:** 10.1038/s41598-020-69504-y

**Published:** 2020-07-31

**Authors:** Ecaterina Stribiţcaia, Charlotte E. L. Evans, Catherine Gibbons, John Blundell, Anwesha Sarkar

**Affiliations:** 1 Food Colloids and Bioprocessing Group, School of Food Science and Nutrition, University of Leeds, Leeds, LS2 9JT UK; 2 Nutritional Sciences and Epidemiology Group, School of Food Science and Nutrition, University of Leeds, Leeds, LS2 9JT UK; 3 Appetite Control and Energy Balance Group, School of Psychology, University of Leeds, Leeds, LS2 9JT UK

**Keywords:** Biomaterials, Gels and hydrogels, Soft materials, Rheology, Psychology, Human behaviour, Risk factors

## Abstract

Obesity is one of the leading causes of preventable deaths. Development of satiety-enhancing foods is considered as a promising strategy to reduce food intake and promote weight management. Food texture may influence satiety through differences in appetite sensations, gastrointestinal peptide release and food intake, but the degree to which it does remains unclear. Herein, we report the first systematic review and meta-analyses on effects of food texture (form, viscosity, structural complexity) on satiety. Both solid and higher viscous food reduce hunger by − 4.97 mm (95% confidence interval (CI) − 8.13, − 1.80) and − 2.10 mm (95% CI − 4.38, 1.18), respectively compared to liquid and low viscous food. An effect of viscosity on fullness (95% CI 5.20 (2.43, 7.97) and a moderate effect of the form of food (95% CI − 26.19 (− 61.72, − 9.35) on food intake were noted. Due to the large variation among studies, the results should be interpreted cautiously and modestly.

## Introduction

Obesity is an escalating global epidemic that falls in the spectrum of malnutrition and is associated with substantial morbidity and mortality consequences. In addition to obesity-induced physical disabilities and psychological problems, excess weight dramatically increases a person’s risk of developing chronic non-communicable diseases, such as cardiovascular diseases^1^, cancers^2^ and diabetes^3^. For the first time in the human history, the population with obesity (body mass index, BMI ≥ 30 kg/m^2^) and overweight (BMI ≥ 25 kg/m^2^) has surpassed that of the population with underweight^4^ with current estimation of 1.9 billion adults with overweight globally, of which 650 million are obese^5^. Medical treatment of obesity is currently limited to drug administration and bariatric surgery. The latter carries significant post-operative risks^6^ and even after the surgery, sustained weight loss can only be achieved through well-designed nutritional interventions. Hence, there is an immense need for applying nutritional prevention strategies to change the current “obesogenic” food environment to become more “leanogenic^7^”.

Weight gain is described as an imbalance between the dietary energy intake and energy expenditure^8^. In other words, to maintain a healthy weight, it is required that the quantity of energy consumed matches the quantity of energy expended. Hence, one promising approach adopted by food scientists, nutritionists and psychologists has been to design or optimise food to achieve satiety (that suppresses appetite for longer periods after consumption)^7^, because this directly leads to a reduction in dietary energy intake and at the same time reduces the impact of sensations of hunger on motivation.

One way to conceptualise appetite control is to consider the Satiety Cascade^9,10^. ‘Satiation’ and ‘satiety’ are two distinct terms with the satiety cascade which are often erroneously used as synonyms when referring to different aspects of appetite control. Satiation describes within-meal inhibition and can be said to determine meal size and bring a particular eating episode to an end. On the other hand, satiety is known to be associated with the inter-meal period, through the suppression of hunger and the inhibition of further eating. Satiety is most commonly measured through both subjective appetite ratings such as, hunger, fullness, desire to eat, prospective food consumption (how much people think they could eat) and thirst, whilst satiation can be measured through meal size—that is through food intake^11^.

**Figure 1:**
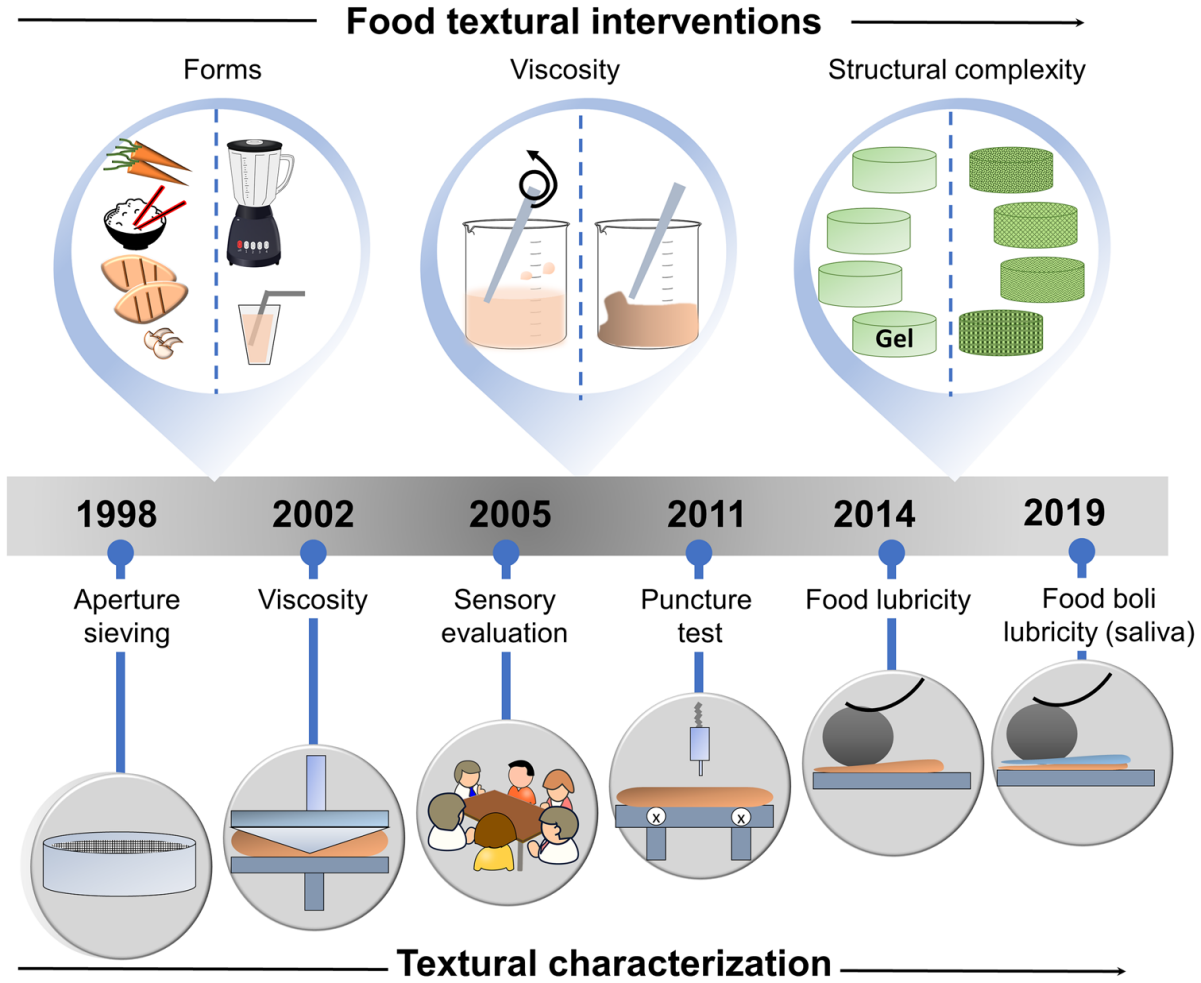
Key milestones in research timeline of food textural manipulations for achieving satiety and the quantitative techniques used to measure food texture.

The current literature on satiety suggests that ‘food texture’ should be an important factor in the control of satiation, satiety, and daily caloric intake. Over the years, the strategy of using food textural manipulations has evolved enormously to the assessment of satiety (see Box 1 and Fig. 1). In addition, various gut peptides, such as ghrelin also known as ‘hunger hormone’^12^, cholecystokinin (CCK), glucagon-like peptide (GLP-1), peptide YY (PYY) are considered to be involved in the regulation of appetite and satiety signalling^13^. Ghrelin is known to increase during fasting and decrease after food intake whereas^12^ GLP-1, CCK and PYY are reduced during fasting periods and released into the circulation after a meal^14^. CCK is also believed to play a role in satiation by reducing food intake^15,16^.

Considering the topical nature of this field, there have been excellent systematic reviews and meta-analyses on appetite control focusing mainly on the intrinsic aspects of eating such as, the effect of chewing^30^, eating rate^31^ or oral processing^32^, which involve physical and physiological aspects of eating and are closely related to an individual’s behaviour. In addition, a meta-regression was conducted on the effects of the time interval between preload and next meal on energy compensation with additional investigation on the effects of physical forms of the preload on energy compensation^33^. The key finding was that the compensatory behaviour decreases faster over time after consumption of semi-solid and solid foods compared to that of liquid products, therefore, suggesting that semi-solids and solids have a greater satiating effect than that of liquids. Also, elegant narrative reviews on the effect of food forms i.e. physical state of food on appetite and energy balance^34^ and impact of food texture and oral processing on satiation/ satiety^35^ are available in the literature reflecting similar conclusions, that semi-solid and solid foods appear to have a stronger satiation response and elicit stronger energy compensation than their liquid counterparts. Along with the previous reviews on this subject, our systematic review adds specific information in regards to the inclusion of more sophisticated and advanced food-texture manipulations to affect satiation and satiety, which is more relevant for future product design and reformulation consideration. Moreover, this study includes the first meta-analysis to quantify the effects of food form and viscosity on hunger, fullness and subsequent food intake. Such details are crucial to allow food researchers and industries to focus on the most appropriate aspects of textural and structural manipulations for rationally designing the next generation foods with ‘just-right texture’. Therefore, studying the precise effects of food texture on appetite control and food intake is very relevant in designing foods with targeted satiety-enhancing properties, and to contribute to the nutritional management of the global pandemic of overweight and obesity.

Here, we report the first systematic review and meta-analysis that aims to investigate the effect of food texture from an external perspective, i.e. how the manipulation of food, its physical state (texture and structure) can impact satiety. The objectives were to understand the influence of food texture on appetite control, including appetite ratings, such as hunger, fullness, desire to eat, thirst, prospective food consumption (how much food participants thought they could eat), food intake, and gut peptides, such as ghrelin, GLP-1, PYY and CCK. We hypothesize that higher textural characteristics (solid form, higher viscosity, higher lubricity, higher degree of heterogeneity, etc.) would lead to greater suppression of appetite and reduced food intake. In this systematic review, the term ‘form of food’ refers to the physical state of food i.e. liquid, solid, semi-solid throughout the entire manuscript.

Box 1: History of food texture interventions in satiety trials The field of ‘food texture-satiety’ was initiated by manipulation of physical forms of food i.e. solid versus liquid or versus semi-solid. In the 1990s, this was achieved by using foods naturally available in different forms, such as whole vegetables and/or meat versus pureed vegetables and/or meat. The techniques used often included blending a solid food resulting in a pureed texture or other kitchen-based food processing techniques, such as boiling, chopping etc.^17,18^. Initially, for instrumental measurements of those texture generated, Santangelo et al.^19^ used a simple 4 mm^2^ aperture sieve to clearly define which food was solid and which one was liquid. Later, the focus on textural intervention shifted to specifically altering the viscosity of food by using different dietary fibres (polysaccharides) to thicken, such as alginate^20^, locust bean gum^21^, or guar-gum^22^ and terms used to describe those textures ranged from 'low viscosity' to 'high viscosity'. At the beginning of 2000, change in viscosity was measured for the first time for use in a satiety trial by Mattes and Rothacker (2001)^23^ using a spindle. The solid food texture was measured using puncture stress^24^ to determine firmness. Besides measurements by instruments, sensory evaluation of food determined by untrained^25^ or trained panels^20^ allowed defining food texture in consumer terms, such as ‘thin’ or ‘thick’. With the field evolving, the texture of food manipulations was more precisely measured in its viscosity and firmness using sophisticated rheological instruments.

A shift in focus occurred a decade later with more attention being given to the structural complexity of food, and to satiety studies using gel-based model foods with precise control over the texture; such gels avoid any emotional association with real food. For instance, Tang et al. (2016)^26^ and Larsen et al. (2016)^27^ were the first ones to use model foods i.e. hydrocolloid based gels with various inclusions to create different levels of textural complexity or in other words higher degree of heterogeneity and assess the relationships between the gels and satiety. Besides classical rheological measurements, McCrickerd et al. (2014)^28^ and Krop et al. (2019)^29^ measured the lubricity of foods without or with simulated saliva (food boli i.e. food and simulated saliva mixture), respectively, using a Mini Traction Machine tribometer. Such differentiation in the lubricity of hydrogels was used for the first time by Krop et al. (2019)^29^ to see their effects on snack intake.

## Methods and materials

This review was registered on the International Prospective Register of Systematic Reviews (PROSPERO) using the Registration Number: CRD42019128434.

### Eligibility criteria

#### Participants

Studies with healthy adults (≥ 18 years old) with a normal weight (BMI = 18.5–24.99 kg/m^2^) were included. Studies involving unhealthy, obese population (obesity is considered a medical condition)^5^ or involving patients suffering from other medical conditions, children (< 18 years old) and elderly (> 60 years old) population were excluded.

#### Interventions

Interventions included any study that manipulated the food texture externally i.e. ranging from varying food forms to its complexity (see Table 1). Only those studies with a fixed-portion preload design i.e. studies where participants were given a fixed amount of preload followed by collection of appetite ratings and/or food intake measurements at a certain interval period of time were included. Any study that involved manipulation of the intrinsic behaviours such as chewing, eating rate have been excluded^36^. Studies that investigated the effects of fibre/fiber or fibre dose and its physiological effects other than manipulation of texture^37,38^ or effects of sugar^39^, studies that compared only high energy density with low energy density with ambiguous reference to the texture were excluded^40^. Also, studies that failed to make the link between food texture and appetite control or food intake or gut peptides, were excluded, for instance studies which assessed expected satiety^41^. Studies that measured food intake following an ad libitum experimental intervention were excluded too^28,42–44^. Likewise, studies that included any cognitive manipulation^45^, a free-living intervention or partial laboratory intervention designs^46^ were excluded to reduce heterogenity in study design. A detailed information on the search terms is given in Supplementary Table S1.

**Table 1:** Food texture parameters of the interventions/preloads as described across studies.

Parameters	Comparison factors	
Form	Liquid	Solid/semi-solid
Viscosity	Low viscous/ thin	High viscous/ thick
Lubricity	Low lubricity	High lubricity
Homogeneity	Homogeneous	Heterogeneous
Structural complexity	Low complexity	High complexity
Aeration	Non-aerated	Aerated

### Meta-analysis

Articles were assessed for eligibility for inclusion in a meta-analysis. All outcomes were assessed for suitability for pooled analysis. A minimum of 3 studies were needed for each meta-analysis. Studies with no reported measure of variation such as standard deviation or standard error were excluded. If data were insufficient to allow inclusion in the meta-analysis, authors were contacted for retrieving the information^47^. Appetite is usually measured on a 100 mm visual analogue scale (VAS)^48^. Where 9, 10 or 13 point scales were used to measure appetite ratings, these scales were converted into a 100 point scale, so that the appetite ratings were comparable^32^. Food intake is measured in either weight (g) or energy (kcal or kJ). The given values were converted to kcal to allow comparison across the studies. For appetite ratings, available data from the medium follow up period (60 min after preload consumption) were extracted for synthesis in meta-analyses. Where meta-analysis was possible, mean differences were calculated to account for variable outcome measures for each comparison, using the generic inverse variance method, in a random-effect meta-analysis model^47^. Stata15 software was used for all analysis. Heterogeneity was assessed using the I^2^ statistic, where I^2^ values of < 50% were considered as acceptable levels of heterogeneity. Funnel plots were presented to assess small study publication bias. Where such data pooling was not possible, findings were narratively synthesised and reported according to the outcomes^47^.

Note, in the Sect. 3.5 on Meta-analysis, *p* values in the text refer to the effect size of food texture on the outcome, while *p* values on the figures refer to the degree of heterogeneity (I^2^).

## Results

The literature search yielded 29 studies that met the inclusion criteria of this systematic review. All studies measured subjective appetite ratings such as hunger, fullness, desire to eat and/or prospective consumption (i.e. how much food participants thought they could eat). Of these, 19 measured subsequent food intake and eight measured gut peptide responses.

### Study selection

**Figure 2:**
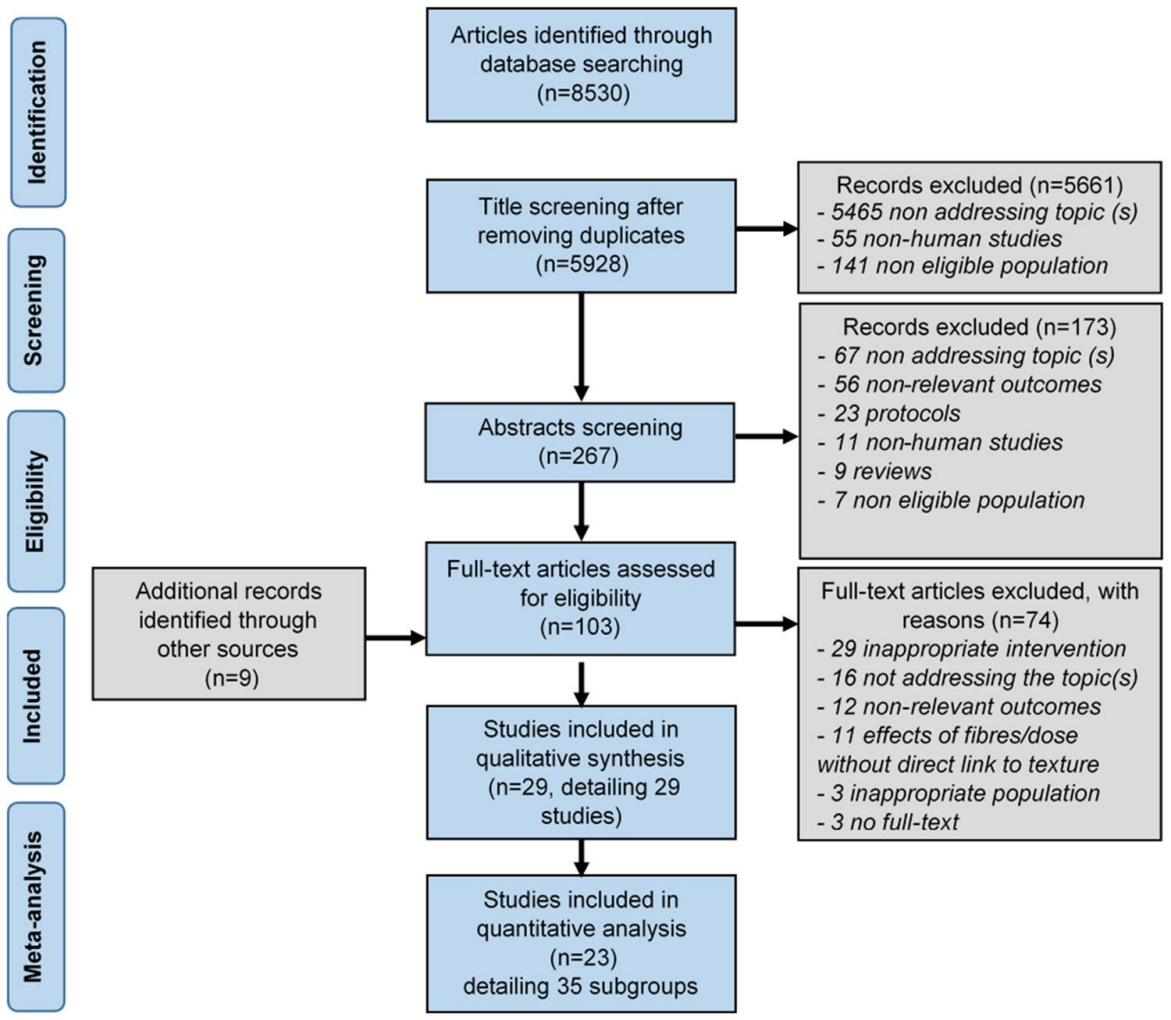
PRISMA flow-chart of the study selection procedure.

The study selection was conducted in several phases following the checklist and flowchart of the PRISMA (Preferred Reporting for Systematic Reviews and Meta-Analyses) guidelines^49^ as shown in Fig. 2. Initially, a total number of 8,530 articles were identified using literature search in the afore-mentioned six electronic databases.

After removing the duplicates (2,602), the remaining 5,928 titles were screened by the first author (ES) based on their relevance to this review. Firstly, 5,661 studies were excluded based on the PICOS (Population, Intervention, Comparison, Outcome, and Setting) criteria i.e. articles involving animal studies (55), or clinical studies involving patients and/or children or elderly population (141) were excluded. Additionally, articles not addressing the topic of interest were excluded (5,465).

The articles were taken to the next phase where 267 abstracts were screened by ES and AS, resulting in the exclusion of an additional 173 articles (67 articles had no relevance to the topic (s) of systematic review, 56 had non-relevant outcome measures, 23 were new or validation of existing protocols, 11 were non-human studies with additional 7 being non-eligible population and 9 were reviews without any original data). A total of 103 full-text articles, including 9 articles that have been identified through supplementary approaches (e.g. manual searches of reference list of pre-screened articles) were equally divided and screened independently by ES, AS and CG. After a mutual agreement, articles with inappropriate interventions and designs (e.g. eating rate, chewing, free-living design, that included any cognitive manipulation, effect of sugar and fats on satiety and appetite) (n = 29) were excluded. In addition, studies not addressing the topic of interest (n = 16) or having non-relevant outcomes (n = 12) were not considered. Articles where the effects of fibres or dosage of fibre were studied without any direct relevance to textural manipulation (n = 11), articles with no full-text (n = 3) or non-relevant population (n = 3) were also eliminated. To sum it up, a total of 29 articles were included for qualitative synthesis.

### Study characteristics

Relevant information such as study design, participant gender, type of intervention on texture manipulation, methods of analysing/measuring the texture of food as well as study outcome on appetite ratings, gut peptides and food intake was extracted from the 29 included studies (Table 2).

**Table 2:** Characteristics of studies included in the systematic review.

Reference	Participants		Study design	Food form/texture manipulation		Food/texture measurements	Outcomes measurements					
	n	Gender M/F		Type of food	Type of manipulation		Appetite method	Effect appetite	Food intake method	Effect food intake	Gut peptides method	Effect gut peptides
Camps, Mars, de Graaf and Smeets (2016)^21^	15	15/0	Randomized, cross-over, within-subjects design, sample size power calculation	**Thick versus thin** Shakes	**Fibre added** Locust bean gum	Viscosity Sensory	VAS-100 mm	Fullness ↑ in thick condition compared to thin one	Ad libitum	No difference in food intake between thick and thin conditions	N/A	N/A
Clegg, Ranawana, Shafat and Henry (2013)^63^	12	6/6	Randomized, cross-over, within-participants, non-blind design	**Solid versus chunky versus smooth** Rice, vegetable and chicken	**Blending** Half ingredients blended + rice added All ingredients blended together	N/A	VAS-100 mm	Fullness ↑ in smooth condition compared to solid one	N/A	N/A	N/A	N/A
Dong, Sargent, Chatzidiakou, Saunders, Harkness et al. (2016)^55^	24	17/7	Randomized, cross-over, within-subjects, double-blind design, sample size power calculation	**Liquid versus semi-solid versus solid** Oranges	**Whole + fibre added** Orange juice Orange juice with orange pomace fibre added Whole oranges chopped	Viscosity	VAS-100 mm	Fullness ↑ in semi-solid and solid condition compared to liquid one	N/A	N/A	N/A	N/A
Flood and Rolls (2007)^64^	60	30/30	Randomized, cross-over, within-subjects design, sample size power calculation	**Solid versus chunky versus chunky-purred versus purred** Broth and vegetables	**Blending** Ingredients combined into a chunky soup All ingredients blended together	Viscosity	VAS-100 mm	No difference in appetite ratings between preloads	Ad libitum	No difference in food intake between conditions	N/A	N/A
Flood-Obbagy and Rolls (2009)^65^	58	30/28	Randomized, cross-over, within-subjects design, sample size power calculation	**Solid versus semi-solid versus liquid** Apples	**Blending + pectin added** Slices, pureed and apple juice with fibre	N/A	VAS-100 mm	Hunger ↓ and fullness ↑ in solid and semi-solid condition compared to liquid one	Ad libitum	Food intake ↓ after solid and semi-solid consumption compared to liquid	N/A	N/A
Hogenkamp, Stafleu, Mars and de Graaf (2012)^53^	27	9/18	Randomized, cross-over, within-subjects design	**Liquid versus semi-solid** Gelatine	**Fibre added** Starch	Sensory evaluation (n = 20)	10-point scale	Fullness ↑ in semi-solid condition compared to liquid one	Ad libitum	No difference in food intake in regard to texture	N/A	N/A
Hogenkamp, Mars, Stafleu and de Graaf (2012)^52^	53	12/41	Randomized, cross-over, within-subjects design, sample size power calculation	**Liquid versus semi-solid** Milk-based products	**Fibre added** Starch	Sensory evaluation (trained, n = 29)	VAS-100 mm	Hunger ↓ and fullness ↑ in semi-solid condition compared to liquid one	N/A	N/A	N/A	N/A
Juvonen, Purhonen, Salmenkallio-Marttila, Lahteenmaki et al. (2009)^67^	20	4/16	Randomized, cross-over, within-subject, single-blind, design	**Low viscous versus high viscous** Oat bran beverages	**Fibre added** Beta-glucanase enzyme	Viscosity	VAS-100 mm	Satiety ↑ in low viscous condition compared to high viscous one	Ad libitum Food records	No difference in food intake between conditions after ad libitum meal Food intake ↑ after low viscosity condition when energy intake of ad libitum and during the rest of the day was combined	Ghrelin, CCK, GLP-1 and PPY	CCK, GLP-1, PYY ↑ and ghrelin ↓ in low viscous condition compared to high viscous one
Juvonen, Karhunen, Vuori, Lille, Karhu, Jurado-Acosta, Laaksonen et al. (2011)^24^	8	8/0	Randomized, cross-over, within-subject design	**High viscous versus low viscous** Milk protein based	**Fibre added** Casein and transglutaminase-treated casein	Puncture test (firmness) Viscosity	VAS-100 mm	Fullness ↑ in gel condition compared to low and high viscous ones	N/A	N/A	GLP-1 and PYY	CCK↑ in high and low viscous condition compared to rigid gel
Krop, Hetherington, Miquel and Sarkar (2019)^29^	55	16/39	Randomized, between-subject design	**High lubricity versus low lubricity** Hydrogels	**Gelling agents** *k-*carrageenan and sodium alginate added	Compression test Viscosity Friction Lubrication Sensory evaluation (trained, n = 11)	VAS-100 mm	No difference in appetite ratings between preloads	Ad libitum	Food intake ↓ after high lubricating gel consumption compared to medium and low lubricating ones	N/A	N/A
Laboure, van Wymelbeke, Fantino and Nicolaidis (2002)^25^	12	12/0	Cross-over, within-subject design Randomization unclear	*Product 1* **Semi-solid versus liquid** Vegetables with beef *Product 2* **Solid versus liquid** Rusk	*Product 1* **Blending** *Product 2* **Toasted or dissolved** in unskimmed chocolate milk	Sensory evaluation (unpublished results)	VAS-100 mm	No difference in appetite ratings between preloads	Ad libitum	No difference in food intake between conditions	N/A	N/A
Larsen, Tang, Ferguson and James (2016)^27^	26	N/A	Randomized, cross-over, within-subjects design	**High complexity versus low complexity** Gelatine agar gels	**Fibre added** Gelatine-agar + ground poppy and sunflower seeds	Sensory evaluation (untrained, n = 20)	VAS-10 cm	No difference in appetite ratings between preloads	Ad libitum	Food intake ↓ after high complex gel condition	N/A	N/A
Marciani, Hall, Pritchard, Cox, Totman, Lad, Hoad, Foster, Gowland and Spiller (2012)^56^	22	13/9	Randomized, cross-over, within-subjects design, sample size power calculation	**Solid versus liquid** Chicken and vegetables	**Blending** All ingredients blended together	Viscosity	VAS 1 to 10	Hunger ↓ in soup condition compared to solid–liquid one	N/A	N/A	N/A	N/A
Martens, Lemmens, Born and Westerterp-Plantenga (2012)^58^	10	10/0	Randomized, cross-over, within-subject, design, sample size power calculation	**Solid versus liquid** Peaches	**Blending** Whole peeled peached or blended	N/A	VAS-100 mm	No difference in appetite ratings between preloads	N/A	N/A	Ghrelin	No difference in ghrelin between conditions
Martens, Lemmens, Born and Westerterp-Plantenga (2011)^57^	10	10/0	Randomized, cross-over, within-subjects design, sample size power calculation	**Solid versus liquid** Chicken	**Blending** Whole steamed chicken or blended	N/A	VAS-100 mm	Hunger ↓ in solid condition compared to liquid one	N/A	N/A	Ghrelin	No difference in ghrelin between conditions
Mattes (2005)^17^	31	13/18	Cross-over, within-subject design Randomization unclear	**Solid versus soup** Apple Chicken breast peanuts	**Blending** Whole ingredients or blended	Viscosity	13-point bipolar category	Hunger ↓ in beverage compared to soup and solid conditions Fullness ↑ in soup and solid conditions compared to beverage one	Food records Unclear if it was served ad libitum or fixed	Energy intake ↓ after soups consumption compared to sild one after 24 h	N/A	N/A
Melnikov, Stoyanov, Kovacs, Arnaudov, de Groot, Schuring, Wiseman, Mela and Peters (2014)^54^	24	3/21	Randomized, cross-over, within-subjects design, sample size power calculation	**Liquid versus aerated** Liquid drink	**Aerated** N_2_O incorporated	Stability	VAS-100 mm	Hunger ↓ and fullness ↑ in aerated condition compared to non-aerated one	N/A	N/A	N/A	N/A
Mourao, Bressan, Campbell and Mattes (2007)^50^	60	30/30	Between-subjects design, sample size power calculation Randomization unclear	**Beverage versus solid** Cheese, watermelon fruit and coconut meat	**No texture manipulation** Whole or bought juice	N/A	VAS-100 mm	No difference in appetite ratings between preloads	Food records	Food intake ↓ after solid consumption compared to liquid one	N/A	N/A
Santangelo, Peracchi, Conte, Fraquelli and Porrini (1998)^19^	8	8/0	Randomized, cross-over, within-subject design	**Solid–liquid versus homogenized** Vegetables, cheese, croutons and olive oil	**Blending** Whole ingredients or homogenized	Aperture sieve	100-mm fixed point scale	Satiety ↑ in homogeneous condition compared to solid one	N/A	N/A	CCK	No difference in CCK between conditions
Solah, Kerr, Adikara, Meng, Binns, Zhu, Devine and Prince (2010)^20^	33	N/A	Randomized, cross-over, within-subject, single-blinded design	**High viscosity versus low viscosity** Water based drinks	**Fibre added** Alginate and protein in water	Viscosity Sensory evaluation (trained, n = 33)	VAS-100 mm	Hunger ↓ in high viscous condition compared to low viscous one	N/A	N/A	N/A	N/A
Tang, Larsen, Ferguson and James (2016)^26^	38	22/16	Randomized, cross-over, single-blind, design	**Low complexity versus medium complexity versus high complexity** Gelatine agar gels	**Fibre added** Gelatine-agar + ground poppy and sunflower seeds	Puncture stress Sensory	VAS-100 mm	Hunger ↓ and fullness ↑ in high complex gels compared to low complex ones	Ad libitum	Food intake ↓ after high complex gels consumption compared to low complex ones	N/A	N/A
Tournier and Louis-Sylvestre (1991)^18^	13	7/6	N/A	**Liquid versus solid** Vegetables and tomato juice	**Blending + fibre** All ingredients mashed and added gelatine	N/A	100-mm lines	No difference in appetite ratings between preloads	Ad libitum Food records	No difference in food intake between conditions	N/A	N/A
Tsuchiya, Almiron-Roig, Lluch, Guyonnet and Drewnowski (2006)^66^	32	16/16	Randomized, cross-over, within-subjects design, sample size power calculation	**Semi-solid versus liquid versus beverage** Peaches	**Blending** Peach pieces in yogurt, the same yogurt homogenized	Sensory evaluation No data shown	9-point category scale	Fullness ↑ in semi-solid and liquid condition compared to beverage one	Ad libitum	No difference in food intake between conditions	N/A	N/A
Wanders, Feskens, Jonathan, Schols, de Graaf and Mars (2014)^59^	29	29/0	Randomized, cross-over, within-subjects, single-blind design, sample size power calculation	**Gels versus capsules versus liquids** Mixture of soft cheese, milk, apple juice and strawberry syrup	**Fibre added** Pectin	Viscosity	VAS-100 mm	Hunger ↓ and fullness ↑ in gel condition compared to capsules and liquid ones Fullness ↑ in capsules condition compared to liquid one	Ad libitum	Energy intake ↓ after capsules consumption compared to liquid condition	N/A	N/A
Yeomans, Wickham, Lundholm and Chambers (2016)^60^	23	23/0	Counterbalanced, within-subjects, design	**Thin **(low sensory) **versus thick** (enhanced sensory) Fruit yogurt beverages	**Fibre added** Tara-gum added	Viscosity and lubrication (stated elsewhere)	VAS-100 mm	Hunger ↓ in thick condition compared to thin one	Ad libitum	No difference in food intake in regard to texture	CCK	No difference in CCK between conditions regards to texture
Yeomans, McCrickerd, Brunstrom and Chambers (2014)^51^	48	48/0	Randomized, between-subjects design	**Thin **(low sensory) **versus thick** (enhanced sensory) Mango and peach yogurt beverages	**Fibre added** Tara-gum added	Sensory evaluation (untrained, n = 24)	VAS-100 mm	Hunger ↓ and fullness ↑ in thick condition compared to thin one	Ad libitum	No difference in food intake in regard to texture	N/A	N/A
Zhu, Hsu and Hollis (2013)^22^	15	15/0	Randomized, cross-over, within-subjects design	**Standard viscosity versus high viscosity** Chocolate pudding	**Fibre added** Guar-gum added	Viscosity	VAS-100 mm	Hunger ↓ and fullness ↑ in high viscous condition compared to low viscous one	Ad libitum	No difference in food intake between conditions	N/A	N/A
Zhu, Hsu and Hollis (2013)^61^	19	19/0	Randomized, cross-over, within-subjects design	**Liquid–solid versus liquid** Vegetables	**Blending** Whole pieces of vegetables in chicken broth or all blended	Viscosity	VAS-100 mm	Fullness ↑ after liquid condition compared to solid one	Ad libitum	No difference in food intake between conditions	CCK Ghrelin	CCK ↑ in liquid condition compared to solid one No difference in ghrelin between conditions
Zijlstra, Mars, de Wijk, Westerterp-Plantenga, Holst and de Graaf (2009)^62^	32	12/20	Randomized, within-subjects cross-over design	**Liquid versus semi-solid** Milk based products	**Fibre added** Starch added	Viscosity Sensory	10-point category scale	Fullness ↑ in semi-solid condition compared to liquid one	Ad libitum	No difference in food intake between conditions	CCK and GLP-1	No difference in CCK between conditions

#### Study design

Many included studies adopted a within-subject design, with the exception of three which used a between-subject design^29,50,51^.

#### Participants

A total of 817 participants were included in the qualitative synthesis with age ranging from 18 to 50 years (mean age 24.7 years), with the exception of two studies not reporting the participants’ age^18,27^. Ideally, studies should have an equal ratio of men and women, however, in five studies more women were included than men^17,29,52–54^. On the other hand, a number of studies included more men than women^26,55,56^. Moreover, in twelve studies men only were included^19,21,22,24,25,51,57–62^. No study included only females and two studies did not mention gender ratio^20,27^. Only five studies had an equal male/female ratio^50,63–66^. All studies selected participants within a healthy BMI range. Mourao et al.^50^ included both lean and obese participants. However, for this systematic review, the results of lean subjects only were included. In most studies, participants with dietary restrictions or dramatic weight change were specifically excluded as well as those who reported high levels of dietary restraint (11 out of 29) as assessed by either the Dutch Eating Behaviour Questionnaire (DEBQ) or the Three Factor Eating Questionnaire were excluded. Only one study was double-blinded^55^ and 14 studies used cover stories to distract participants from the real purpose of the study. In only twelve of the studies, a power calculation was used to determine the number of participants needed to find a significance difference^21,50,52,54–59,64–66^.

#### Intervention

In 16 studies^17,18,25,50,52,53,55–58,61–66^, manipulations of food forms that were included consisted of liquid versus solid or liquid versus semi-solid or semi-solid versus solid, and included chunky and pureed food. Food consisted mainly of vegetables, fruit, meat and beverage (fruit juices) and texture was manipulated by blending the food. Eight studies^20,21,24,51,59–61,67^ investigated the effect of viscosity, such as low viscosity/ (sensorially termed as ‘thin’) versus high viscosity/ (sensorially termed as ‘thick’), and the texture was manipulated by adding fibres such as, starch, tara-gum, locust-beam gum, alginate, guar-gum, casein and pectin to food products, such as milk products or fruit juices. Two studies^26,27^ examined the effect of structural complexity, such as low complexity versus high complexity, and the intervention consisted of model foods i.e. hydrogels enclosing various layers and particulate inclusions such as poppy and sunflower seeds. One study^19^ looked at the homogenization of food, one at the aeration of food incorporating N_2_O into a liquid drink^54^ and one study assessed the effect of gels with different lubricity (low vs medium vs high lubricity) using *κ*-carrageenan and alginate to manipulate the texture^29^.

#### Food texture measurements

Nineteen studies measured food texture instrumentally, of which 14 assessed viscosity^17,20–22,24,29,55,56,59–62,64,67^, two measured lubricity indirectly by measuring friction coefficients^29,60^, one measured foam volume as a function of time^54^ and one used aperture sieving^19^. Eleven studies assessed food texture using sensory evaluation, of which three studies have used a trained panel (11, 29, 33 panellists)^20,29,52^, four studies untrained (20, 20, 24, 32 panellists)^26,27,51,62^, and in two studies it is unclear whether it was a trained or untrained panel (20 panellists)^21,53^. Two studies did not publish or did not show the data^25,66^. The sensory evaluation was carried out by using Quantitative Descriptive Analysis (QDA)^27,29^, modified Texture Profile (TP) and Temporal Dominance of Sensations (TDS)^27^.

Additional information with regards to objective textural manipulation that is characterized by instrumental and sensorial techniques and information on weight and energy density of the intervention, and time to next meal can be found in Supplementary Table S2.

#### Appetite ratings method

All studies used 100 mm visual analogue scale (VAS) or categorical rating scales to assess appetite ratings. The majority of studies that assessed appetite control measured hunger (n = 27), fullness (n = 25), and desire to eat ratings (n = 21). Two studies^19,67^ referred only generally to satiety instead of specifying exactly which appetite ratings were being measured.

#### Food intake measurements

Subsequent food intake was measured using ad libitum meal consumption after the intervention in most of the studies (n = 17), but two studies used a food record method^17,50^.

#### Gut peptides

From the limited number of studies that measured gut peptides (n = 8) using blood plasma samples drawn at baseline and different time points after the intervention, two measured CCK alone^19,60^, two measured ghrelin alone^57,58^ and four studies measured more than one gut peptides GLP-1 and PYY^24^, GLP-1 and CCK^62^, CCK and ghrelin^61^, ghrelin, CCK, GLP-1 and PYY^67^. The gut peptides were mainly assayed using commercial plate-based immunoassay test kits.

### Quality assessment

To assess the quality of studies (n = 29) included in this systematic review, Cochrane’s tool of risk of bias was used^68^ with regards to random sequence generation, allocation concealment and blinding of participants and personnel and it is reported in Supplementary Table S3. One study^55^ reported on all three criteria (random sequence generation, allocation concealment and blinding of participants and personnel), and therefore was included in the low risk-of-bias category. Twenty-five studies reported on one or two criteria and were considered as in the medium risk-of-bias category. And three trials^17,18,25^ did not report clearly on the assessment criteria, therefore were judged within the high risk-of-bias category.

### Narrative synthesis

#### Effect of food texture on appetite control

Of the total studies that measured appetite control (n = 29), 16 found a significant effect of food texture on reducing hunger and increasing fullness ratings. The textural manipulation within these studies ranged from the manipulation of solid-like characteristics to viscosity and to the design of well-characterized model gels with structural complexity (Table 2). For instance, it was noticed that the consumption of solid and/or semi-solid food more strongly suppressed appetite ratings as compared to ratings of liquid food. Flood-Obbagy and Rolls^65^ found that whole apples led to decreased hunger ratings and increased fullness when compared with their liquid counterparts (i.e. apple sauce and juice). These authors argued that the effect of food on satiety was due to the structural form of food itself and the larger volume in case of whole fruit as compared to the liquid versions, even when matched for energy content and weight. Interestingly, these findings were not associated with the amount of fibre as the fibre content was similar across liquid and solid conditions. Similar findings by Hogenkamp et al.^52^ indicated that hunger decreased, and fullness increased in the semi-solid condition compared to the liquid condition. They found that the semi-solid product (comparable with firm pudding) suppressed appetite greater than the liquid product (comparable with very thin custard). The authors related their findings to the triggering of the early stages of the satiety cascade^10^ through cognitive factors and sensory attributes such as visual and oral cues; whereas food forms might not affect the later processes in satiety cascade that are postulated to be governed by post-ingestive and post-absorptive factors^52^.

Foods with high viscosity also appeared to play a key role in appetite suppression compared to food with low viscosity^20,22,51,60^. Aiming to determine the effect of viscosity on satiety, Solah et al.^20^ used low and high viscous alginate-based breakfast drinks, on 33 subjects. It was found that hunger was lower after participants consumed the high viscous alginate drink as compared to those who consumed low viscous ones. The authors speculated that such findings were related to the gastric distention as a result of the ingested gel-forming fibre, although they did not measure the rheological properties of these foods in the gastric situation. In a rather long-term (7 non-consecutive days over a month) study, Yeomans et al.^51^ investigated low (thin) and high (thick) viscous drinks with both low and high energy content, respectively. They found that initially, appetite was suppressed after consuming high viscous foods as compared with those who consumed low viscous foods, corroborating the afore-mentioned effect of viscosity on satiety. They related their findings to a slower gastric emptying rate in the high viscous food. However, after repeated consumption of the drinks with seven non-consecutive days over a month, there were no noticeable differences in satiety between the low and high viscous conditions^51^. Expected satiation was higher for both high energy drinks and lower for both low energy drinks irrespective of the viscosity of the foods. This suggests that in a repeated consumption setting, the effect of viscosity can be negligible.

It is noteworthy that some of the authors relate their findings of increased satiety after consuming high viscous foods to a slower gastric emptying rate, which should be interpreted with some caution. For instance, Camps et al.^21^ directly measured the effect of viscosity on gastric emptying in their study using magnetic resonance imaging (MRI) abdominal scans and found that not only the viscosity of food but also the energy load led to a slow gastric emptying. The preloads in their case were four shakes differing in viscosity (low and high viscosity) measured in perceived thickness using 100-mm VAS scale and also differing in energy content (low/100 kcal and high/500 kcal) consumed within 2 min. The increase in the energy load led to slower gastric emptying over time; it only significantly slowed the emptying under the low-energy-load condition. Therefore, they suggested that viscosity loses its reducing effect on hunger if energy load is increased to a meal size of 500 kcal indicating that viscosity may not always affect the later parts of satiety cascade through delayed gastric emptying route, but contributes to the early parts of satiety cascade via mouth feel and oral residence time.

In addition to form and viscosity, textural complexity has also shown some significant effects on appetite control. However, the term textural complexity is rather poorly defined in the literature. Often it refers to the degree of heterogeneity or inhomogeneity in a food where the preload includes some inclusions, which distinguishes it from a control; the latter having a homogenous texture i.e. without inclusions. This research domain of studying the effects of *so-called* textural complexity on satiety is still in its early infancy. Tang et al.^26^ conducted the first trial on textural complexity (of the preload) (Fig. 1) and demonstrated that hunger ratings decreased when model food gels with higher complexity (i.e. gels layered with particulate inclusions) were served. The authors noticed that higher inhomogeneity in the gels with particle inclusions led to a decrease in hunger and desire to eat, and an increase in fullness ratings, suggesting that levels of textural complexity may have an impact on post-ingestion or post-absorption processes leading to a slowing effect on feelings of hunger.

The technique of aeration, (i.e. incorporation of bubbles in a food) has been also used as a textural manipulation and been shown to have an influence on satiety. Melnikov et al.^54^ found that hunger was lower, and fullness was higher in aerated drinks as compared to the non-aerated counterparts. Although these drinks differed in energy content (low/high energy non-aerated and low/high energy aerated), they demonstrated that such aeration independent of energy content was a promising textural manipulation to suppress appetite. The authors attributed the findings to the effect of the air bubbles on gastric volume leading to the feelings of fullness.

In thirteen studies out of the 29 studies, food texture was reported to have no effect on appetite ratings. This disparity in the results may be associated with the methodology employed. For instance, in several studies^27,50,58^ participants were instructed to eat their usual breakfast at home. Therefore, the appetite level before the preload was not controlled and this might have influenced the appetite rating results. Furthermore, some studies did not conceal the purpose of the study from the participants^18,25^. Thus, participants’ responses might have been biased and could have led to less reliable results^69^. Moreover, Mourao et al.^50^ firstly served an ad libitum meal to participants and then immediately the preload with different textural attributes. As such, the time interval between ad libitum intake and preload may have accounted for variation in outcomes^70^. All these factors may explain the disparities with regards to the effects of food texture on subjective appetite ratings.

#### Effect of food texture on gut peptides

Out of the limited number of studies (n = 8) that included gut peptides measurements; only two^61,67^ studies found an effect of food texture. Contrary to our expectations, Juvonen et al.^67^ found that CCK, GLP-1, PYY increased and ghrelin decreased in low viscous condition compared to high viscous one. The authors speculate that after consuming a high viscous drink, viscosity of the product may delay and prevent the close interaction between the nutrients and gastrointestinal mucosa required for efficient stimulation of enteroendocrine cells and peptide release. The same results were found in regard to food form. Zhu et al.^61^ found that liquid food (pureed liquid–solid soup) resulted in a higher postprandial response of CCK comparing with solid food (whole pieces of vegetables in a chicken broth). They related it to the capacity of CCK to be secreted in the duodenum in response to the presence of nutrients. As such, they suggest that the increase in the surface area of the nutrients due to the smaller particle sizes resulted from the pureeing could stimulate secretion of CCK more potently.

The rest of the studies found no significant effect of food texture (form, viscosity or complexity) on triggering relevant gut peptides. This may be due to the type of macronutrients used in such intervention. For example, intervention in Martens’ et al.^57^ study was high in protein and it is known that proteins are less effective in suppressing ghrelin^13^. Therefore, one may argue that the effect of food texture is only restricted to early stages of satiety cascade rather than later stages, where the type and content of macronutrient might play a decisive role. However, such interpretations might be misleading owing to the limited number of studies in this field. Also, in the majority of studies conducted so far, the biomarkers were limited to one gut peptide, such as CKK^19,60,61^ or ghrelin^57,58^, which provides a selective impression of the effects on gut peptides. Measuring more than one gut peptide could provide richer data and wider understanding of the relationship between food texture and gut peptides, which has yet to be fully evaluated^69^.

#### Effect of food texture on energy intake

Seven out of the total 29 studies found a significant effect of texture on food intake. Food form, such as solid, appeared to play a role in the subsequent food/energy intake. For example, in the study by Flood and Rolls^65^, 58 participants consumed apple segments (solid food) on one day and then apple sauce (liquid food) made from the same batch of apples used in the whole fruit conditions on another day. The preload was controlled for the energy density and consumed within 10 min and the ad libitum meal was served after a total of 15 min. As a result, they found that apple pieces reduced total energy intake at lunch as compared to the apple sauce, therefore suggesting that consuming whole fruits before a meal can enhance satiety and reduce subsequent food intake. Mourao et al.^50^ also confirmed such findings where participants consumed less energy after ingesting solid food form (cheese/watermelon fruit/coconut meat) as compared to the beverage form (milk/watermelon juice/coconut milk). However, it is worth noting that they had a different experimental approach in contrast to the rest of the studies in this systematic review. First, an ad libitum meal was served and then followed by a fixed preload consisting of solid and beverage form with one predominant macronutrient (milk-protein, watermelon-carbohydrate and coconut-fat). The time between ad libitum meal and the preload was not stated; it is only clear that it was served at lunch time. Food records were kept on each test day (for 24 h) to determine energy intake. Despite this different approach, it was demonstrated that solid food led to a lower subsequent energy intake compared with liquid food counterparts. Consequently, this study supports an independent effect of texture on energy intake.

In terms of viscosity, it has been found that higher viscous food can also lead to a reduced subsequent energy intake. This was noted in Juvonen’s et al.^67^ study, where participants consumed two identical, isoenergetic and isovolumic oat bran beverages that differed only in their viscosity (low, < 250 mPa; high, > 3,000 mPas) which was measured instrumentally. Authors reported that the beverage with high-viscosity led to a lower energy intake compared to the low-viscous beverage when energy consumption during the meal consumed ad libitum and during the rest of the test day was combined. Although authors attribute their findings to a slower gastric emptying rate, they did not measure it directly, nor was the effect of viscosity on mouth feel or oral residence time affecting early stages of satiety cascade investigated.

Even with a limited number of studies, textural complexity has been demonstrated to have a clear impact on subsequent food intake. For instance, in the studies of Tang et al.^26^ and Larsen et al.^27^, gels mixed with poppy and sunflower seeds reduced subsequent food intake independently of the oral transit time and energy density, suggesting a sole impact of food texture on food intake.

Interestingly, Krop et al.^29^ also showed a clear effect of texture on reducing subsequent snack intake by using hydrogels (having no energy content or micronutrients) that differed in their textural complexity in terms of their lubricating properties, which was measured both instrumentally and sensorially^71^. These authors related their findings to hydrating and mouth-coating effects after ingesting the high lubricating carrageenan-alginate hydrogels that in turn led to a lower snack intake. Moreover, they demonstrated that it was not the intrinsic chewing properties of hydrogels but the externally manipulated lubricity of those gel boli i.e. gel and simulated saliva mixture that influenced the snack intake. All these reports suggest that there is a growing interest in assessing food texture from a textural complexity perspective*.* This means introducing heterogeneity such as tribological/ lubrication alternation in food to have enhanced satiety and satiation consequences. This strategy needs attention in future satiety trials as well as longer-term repeated exposure studies.

The energy density of the preload across the studies varied from zero kcal^29^ or a modest energy density 40 kcal^26,27^ up to a higher value of – 600–700 kcal^18,19^ (see the Supplementary Table S2). It is noteworthy that the lower the energy density of the preload, the shorter the time interval between the intervention (preload) and the next meal (ad libitum meal). Some of these studies showed an effect of texture on appetite ratings and food intake, with food higher in heterogeneity leading to a suppression of appetite and reduction in subsequent food intake^26,27^. Also, gels with no calories but high in their lubrication properties showed a reduction in snack intake^29^. Contrary to those textures with zero (or modest levels of) calories, those textures high in calories tended to have a larger time gap between the intervention (preload) and the next meal. An interesting pattern observed across these studies employing high calorie-dense studies, is that an effect of texture on appetite ratings was found but no effect on food intake^22,61,66^. Therefore, in addition to the high energy density of the preload, it appears that time allowed between the preload and the next meal is an important methodological parameter.

### Meta-analysis

A total of 23 articles were included in the meta-analysis. Two articles were excluded as data on a number of outcomes were missing^19,50^. Meta-analysis on structural complexity^26,27^, lubrication^29^, aeration^54^ and gut peptides could not be performed due to the limited number of studies that addressed this issue, and therefore a further four articles were excluded. Finally, meta-analysis was performed on the effect of form and viscosity of food on three outcomes: hunger, fullness and food intake. Data from 22 within-subjects and 1 between-subjects trials reporting comparable outcome measures were synthesised in the meta-analyses. These articles were expanded into 35 groups as some studies provided more than one comparison group. In most of the studies (n = 18), appetite was measured on 100 mm visual analogue scale (VAS).

Meta-analyses presenting combined estimates and levels of heterogeneity were carried out on studies investigating form (total of 20 subgroups, 651 participants) and viscosity (total of 15 subgroups, 281 participants) for the three outcomes hunger, fullness and food intake (see data included in the meta-analysis in Supplementary Tables S4a–c). There was an insufficient number of studies to carry out meta-analyses for the ones investigating complexity (n = 2)^26,27^, lubrication (n = 1)^29^, aeration (n = 1)^54^ (total of 4 studies, 103 participants) and gut peptides (total of 8 studies, 130 participants (e.g. 3 studies assessed GLP-1 with available data on 2 studies^24,62^, and 2 studies assessed PYY with available data on 1 study only^24^).

#### Hunger

**Figure 3:**
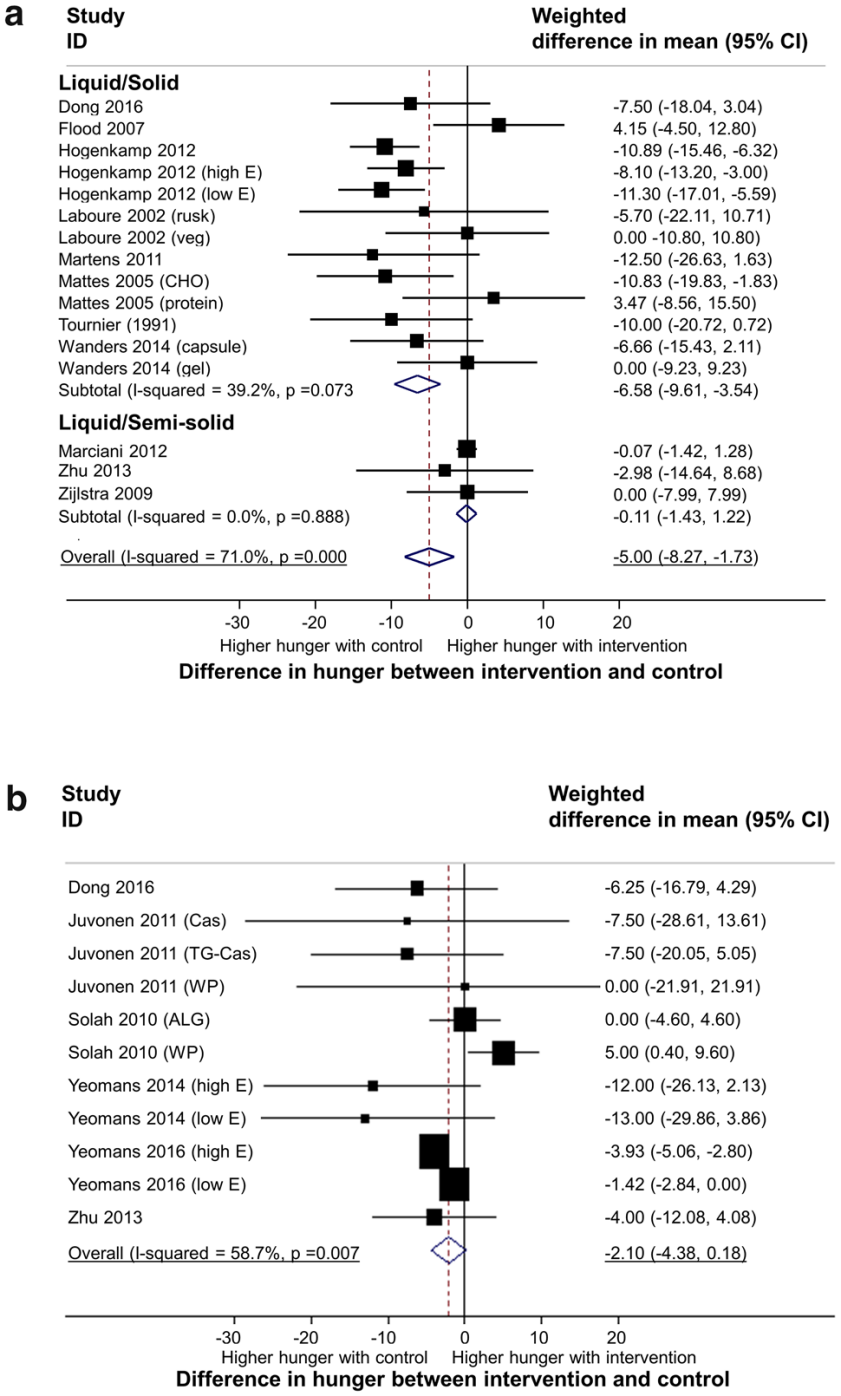
Meta-analysis of effect of food texture on hunger ratings. Pooled estimate of the differences in hunger ratings between intervention and control by food form (liquid/solid; liquid/semi-solid) (**a**) and viscosity (low/high viscous) (**b**), respectively. Available data from the medium follow-up period (60 min after intervention/control) was used for synthesis. The bottom horizontal line denotes 95% CIs. The diamond indicates the overall estimated effect. ID represents the identification.

A meta-analysis of 556 participants, from 16 subgroups based on food form (13 comparing solid with liquid food and 3 comparing semi-solid with liquid food) revealed an overall significant decrease in hunger with the intervention (solid or semi-solid) group of − 5.00 mm (95% confidence interval (CI) − 8.27 to − 1.73, *p* = 0.003, I^2^ = 71%). There was a significant decrease in hunger with solid food of − 6.58 units (95% CI − 9.61 to − 3.54, *p* < 0.001, I^2^ = 39%) however no difference in hunger was seen for comparisons of semi-solid with liquid food (see Fig. 3a). A meta-analysis of 191 participants from 11 subgroups based on viscosity revealed a borderline significant decrease in hunger with higher viscosity food of − 2.10 mm (95%CI − 4.38 to 0.18, *p* = 0.071, I^2^ = 59%) (see Fig. 3b).

#### Fullness

**Figure 4:**
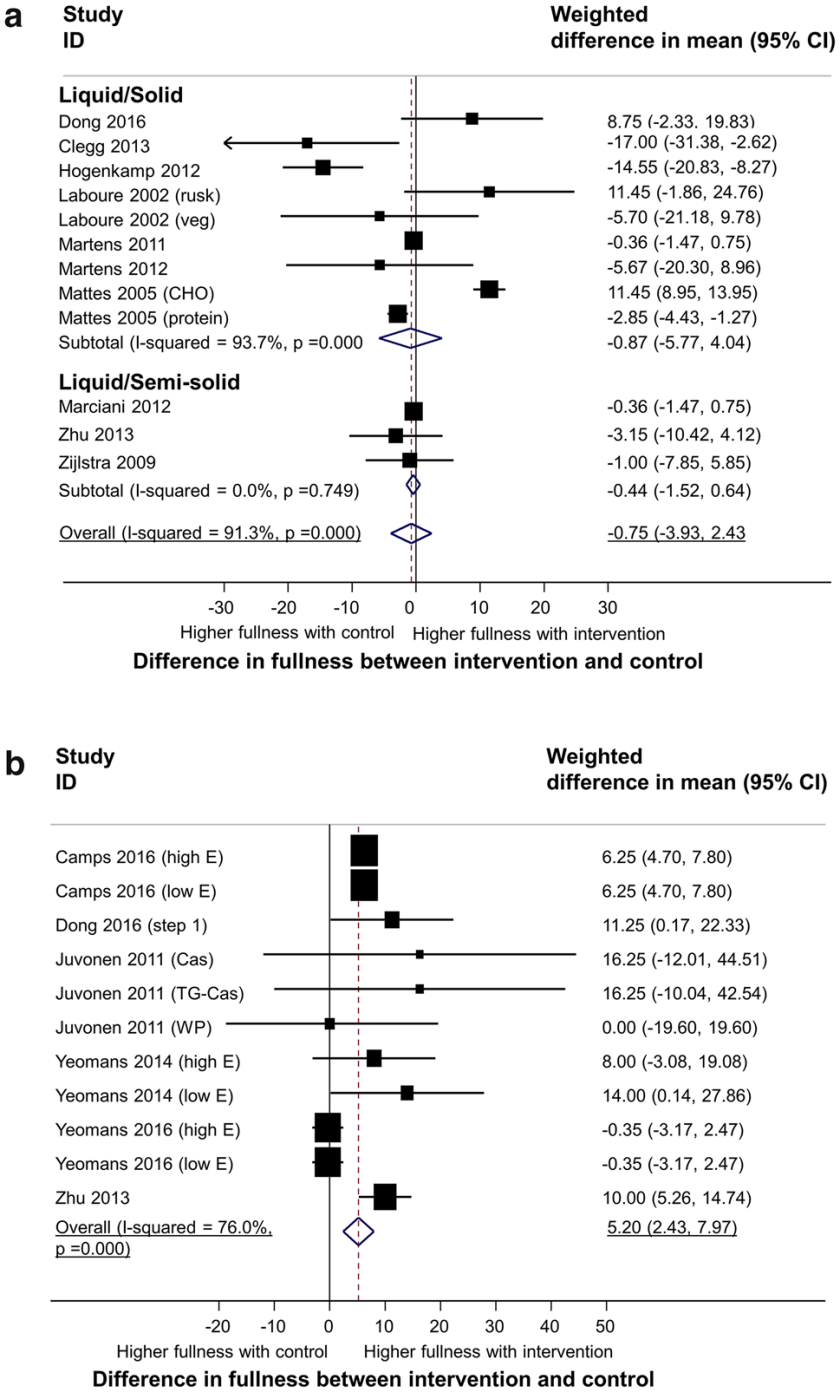
Meta-analysis of effect of food texture on fullness ratings. Pooled estimate of the differences in fullness ratings between intervention and control by food form (liquid/solid; liquid/semi-solid) (**a**) and viscosity (low/high viscous) (**b**). Available data from the medium follow-up period (60 min after intervention/control) was used for synthesis. The bottom horizontal line denotes 95% CIs. The diamond indicates the overall estimated effect. ID represents the identification.

A meta-analysis of 263 participants, from 12 subgroups based on form (9 comparing solid with liquid food and 3 comparing semi-solid with liquid food) revealed no overall difference in fullness between the intervention (solid or semi-solid) group and control group (− 0.75 units, 95% CI − 3.93 to 2.43, *p* = 0.644, I^2^ = 91%). There was no difference in fullness between groups for either of the two subgroups (see Fig. 4a).

A meta-analysis of 155 participants from 11 subgroups based on viscosity revealed an overall significant increase in fullness for higher viscosity food of 5.20 mm (95%CI 2.43 to 7.97, *p* < 0.001, I^2^ = 76%) (see Fig. 4b).

#### Food intake

**Figure 5:**
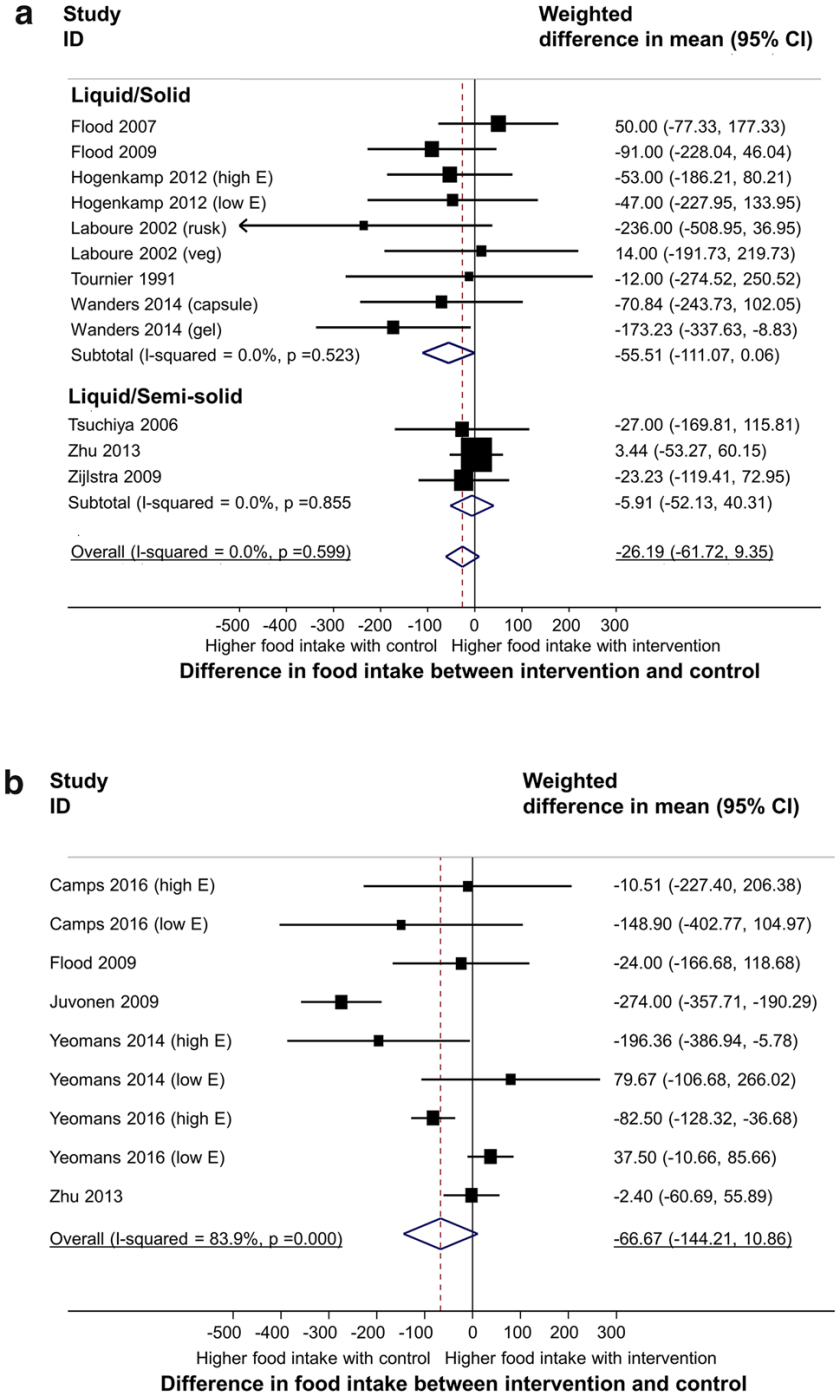
Meta-analysis on effect of food texture on food intake. Pooled estimate of the differences in food intake between intervention and control by food form (liquid/solid; liquid/semi-solid) (**a**) and viscosity (low/high viscous) (**b**). Available data from the medium follow-up period (60 min after intervention/control) was used for synthesis. The bottom horizontal line denotes 95% CIs. The diamond indicates the overall estimated effect. ID represents the identification.

A meta-analysis of 458 participants, from 12 subgroups based on form (9 comparing solid with liquid food and 3 comparing semi-solid with liquid food) revealed no overall difference in food intake with the intervention (solid or semi-solid) group compared with the control group (− 26.2 kcal, 95% CI − 61.7 to 9.4 kcal, *p* = 0.149, I^2^ = 0%) (see Fig. 5a).

There was a borderline significant reduction in food intake for studies comparing solid with liquid food of − 55.5 kcal (95% CI − 111.1 to − 0.1 kcal, *p* = 0.05, I^2^ = 0%) however no difference in food intake was seen for comparisons of semi-solid and liquid food. A meta-analysis of 191 participants from 9 subgroups based on viscosity revealed a non-significant decrease in food intake with higher viscosity food of − 66.7 kcal (95%CI − 144.2 to 10.9 kcal, *p* = 0.092, I^2^ = 84%) (see Fig. 5b). Funnel plots (see Supplementary Figure S1a–c) reveal that there was some evidence of asymmetry and therefore publication bias may be present, particularly for the meta-analyses for hunger.

## Discussion

In this comprehensive systematic review and meta-analysis, we investigated the effects of food texture on appetite, gut peptides and food intake. The hypothesis tested was that food with higher textural characteristics (solid form, higher viscosity, higher lubricity, higher degree of heterogeneity, etc*.*) would lead to a greater suppression of appetite and reduced food intake. In fact, the qualitative synthesis showed that in half of the studies included in this systematic review food texture such as, solid form^17,50,52,53,55,57,62,65,66^, higher viscosity^20–22,24,51,59,60^, higher lubricity^29^, higher degree of complexity/heterogeneity^26,27^ and aerated^54^ food was reported to suppress appetite and reduce food intake. Likewise, the quantitative analysis (meta-analysis) clearly indicated a significant decrease in hunger with solid food compared to liquid food. Also, a significant increase was noted in fullness with high viscous food compared to low viscous food. However, no effect of food form on fullness was observed. Food form showed a borderline significant decrease in food intake with solid food having the main effect.

The main explanation for the varying outcomes could be the methodology applied across the studies which was supported by a moderate to a high heterogeneity of studies in the meta-analysis. Within the preload study designs that were included in the current article, attention should be paid to the following factors that were shown to play an important role in satiety and satiation research: macronutrient composition of the preload, time lapse between preload and test meal, and test meal composition^70^.

Considerable data supports the idea that the macronutrient composition, energy density, physical structure and sensory qualities of food plays an important role in satiety and satiation. There appears to be a hierarchy (protein > carbohydrate > fat) in the extent to which macronutrients can impact satiety and satiation^72,73^. For instance, it has been demonstrated that eating a high-protein and high-carbohydrate preload can lead to a decrease in hunger ratings and reduced food intake in comparison with eating high-fat preload^72^. As such, it is worth noting that interventions across the studies included in this systematic review and meta-analysis differed hugely in terms of macronutrient composition. For example, in some studies the preload food was higher in fat and carbohydrate^25,64^ compared to protein which may be a reason for finding no effect on appetite and food intake. In contrast, where the preload was high in protein^57^, a significant suppression of appetite ratings was observed. Moreover, it is important to highlight that a recent development in the food science community is the ability to create products such as hydrogel-based that do not contain any calories. As these gels are novel products, they are also free from any prior learning or expected postprandial satisfaction that could influence participants. These hydrogels have been proven to have an impact on satiety^26^ and satiation^29^ suggesting there is an effect of food texture alone, independent of calories and macronutrients composition.

An important factor that may also explain variation in outcomes, may be the timing between preload and test meal. It has been argued that the longer the time interval between preload and test meal the lower the effect of preload manipulation^74^. Accordingly, the range of intervals between preload and test meal differed substantially across the studies included in this systematic review: from 10 to 180 min. Studies with a shorter time interval (10–15 min) between preload and ad libitum food intake showed an effect of food texture on subsequent food intake^26,27,29^. In contrast, those studies with a longer time interval, such as Camps et al.^21^, Tsuchiya et al.^66^, Yeoman et al.^51,60^ (90 min) and Tournier et al.^18^ (180 min) found no effect on food intake.

As such, it can be deduced that the effects of texture might be more prominent in studies tracking changes in appetite and food intake over a shorter period following the intervention. In addition, the energy density of the preload is a key factor that should not be discounted when designing satiety trials on food texture. For instance, the lower the energy density of the preload, the shorter the interval between the intervention and next meal should be in order to detect an effect of food texture on satiation as observed by Tang et al.^26^, Larsen et al.^27^, Krop et al.^29^ (see Supplementary Table S2). Therefore, the different time intervals between preload and ad libitum test meal, and a difference in energy densities of the preload can lead to a modification of outcomes, which might confound the effect of texture itself.

The test meals in the studies were served either as a buffet-style (participants could choose from a large variety of foods) or as a single course (food choice was controlled). It has been noticed that in studies where the test meal was served in a buffet style^25,53,66^, there was no effect on subsequent food intake. Choosing from a variety of foods can delay satiation, stimulate more interest in different foods offered and encourage increased food intake^75^ leading to the same level of intake on both conditions (e.g. solid and liquid conditions). In contrast, in studies that served test meal as a single course^26,27,29,67^, the effect of texture on subsequent food intake has been shown as more prominent. Therefore, providing a single course meal in satiety studies may have scientific merit although it might be far from real-life setting.

It was also noticeable that some studies with a larger sample size^17,20,60^ showed less effect of food texture on hunger and fullness in our meta-analysis. Although, it is not possible to confirm the reasons why this is the case we can only speculate it could be due to considerable heterogeneity across the studies. For instance, one of the reasons could be the selection criteria of the participants. Even though, we saw no substantial differences from the information reported in individual studies there may be other important but unreported factors contributing to this heterogeneity. Furthermore, studies with larger sample sizes often have larger variation in the selected participant pool than in smaller studies^76^ which could potentially reduce the precision of the pooled effects of food texture on appetite ratings but at the same time may produce results that are more generalizable to other settings.

Although the meta-analysis showed a clear but modest effect of texture on hunger, fullness and food intake, the exact mechanism behind such effects remains elusive. Extrinsically-introduced food textural manipulations such as those covered in this meta-analysis might have triggered alterations in oral processing behaviour, eating rate or other psychological and physiological processing in the body. However, at this stage, to point out one single mechanism underlying the effect of texture on satiety and satiation would be premature and could be misleading. A limited number of studies have also included physiological measurements such as gut peptides with the hypothesis that textural manipulation can trigger hormonal release influencing later parts of the Satiety Cascade^9,10^. However, with only eight studies that measured gut peptides, of which five failed to show any effect of texture, it is hard to support one mechanism over another. Therefore, more studies are needed especially incorporating physiological measurements in order to understand the whole spectrum of mechanisms underlying an effect of food texture on satiety/satiation.

## Future strategies

Employing food textural manipulations such as increasing viscosity, lubricating properties and the degree of heterogeneity appear to be able to trigger effects on satiation and satiety. However, information about the physiological mechanism underlying these effects have not been revealed by an examination of the current literature. Unfortunately, many studies in this area were of poor-quality experimental design with no or limited control conditions, a lack of the concealment of the study purpose to participants and a failure to register the protocol before starting the study; thus, raising questions about the transparency and reporting of the study results. Future research should apply a framework to standardize procedures such as suggested by Blundell et al.^70^ in order to have more consistent results and to justify a claim for an effect of food on subjective aspects of appetite and food intake.

Also, the recent development of food colloidal approaches to create products/hydrogels with no calories and macronutrients was noted. It is, therefore, crucial to carry out more studies involving these types of well-characterized model foods and see how they may affect satiety and food intake. To date, only one study^29^ has looked at the lubricating capacity of food using hydrogels with no calories which clearly showed the effect of texture alone; eliminating the influence of energy content. As such, a clear gap in knowledge of the influence of food with higher textural characteristics, such as lubrication, aeration, mechanical contrast, and variability in measures of appetite, gut peptide and food intake is identified through this systematic review and meta-analysis.

There are limited number of studies that have assessed gut peptides (ghrelin, GLP-1, PPY, and CCK) in relation to food texture to date. Apart from the measurement of gut peptides, no study has used saliva biomarkers, such as α-amylase and salivary PYY to show the relationship between these biomarkers and subjective appetite ratings. Therefore, it would be of great value to assess appetite through both objective and subjective measurements to examine possible correlations between the two.

Besides these aspects, there are other cofactors that are linked to food texture and hard to control, affecting further its effect on satiety and satiation. To name, pleasantness, palatability, acceptability, taste and flavour are some of the cofactors that should be taken into account when designing future satiety studies. In addition, effects of interactions between these factors such as taste and texture, texture and eating rate etc*.* on satiety can be important experiments that need future attention.

Also, measuring the texture of the food/preload both instrumentally and by sensory procedures, can increase the quality of study design and give more accurate and robust results. This would help to objectively understand the degree of sensorial distinction/ instrumental difference needed between the intervention and the control to have an effect on satiety. For instance, the higher viscous food should have at least 10–100 factor higher viscosity than the control at orally relevant shear rate (i.e. 50 s^−1^) to see some effects of viscosity on satiety. Therefore, objectively characterizing the preloads in the study by both instrumental and sensory terms is important to have a significant effect of texture on satiety.

Furthermore, having a control condition, such as water or placebo condition, will make sure that the effects seen are due to the intervention (preload) and not to some other factors. Also, time to the next meal is crucial. Studies with a low energy density intervention should reduce the time between intervention and the next meal. Also, double-blind study designs should be considered to reduce the biases. Finally, intervention studies with repeated exposure to novel food with higher textural characteristics and less energy density are needed to clearly understand their physiological and psychological consequences, which will eventually help to create the next-generation of satiety- and satiation-enhancing foods.

## Supplementary information

Supplementary information
