# Supplementary material for: Food texture influences on satiety: systematic review and meta-analysis
Source: Sci Rep. 2020 Jul 31;10:12929. doi: 10.1038/s41598-020-69504-y (PMC7395742; doi:10.1038/s41598-020-69504-y)
Supplement: Supplementary file 1 — Supplementary information [file 41598_2020_69504_MOESM1_ESM.docx]

**Food texture influences on satiety: Systematic review and meta-analysis**

*Ecaterina Stribițcaiaª, Charlotte E. L. Evans^ᵇ^, Catherine Gibbonsᶜ, John Blundellᶜ, Anwesha Sarkarª**

ª Food Colloids and Bioprocessing Group, School of Food Science and Nutrition, University of Leeds, Leeds LS2 9JT, United Kingdom

^ᵇ^ Nutritional Sciences and Epidemiology Group, School of Food Science and Nutrition, University of Leeds, Leeds LS2 9JT, United Kingdom

ᶜ Appetite Control and Energy Balance Group, School of Psychology, University of Leeds, Leeds LS2 9JT, United Kingdom

*Corresponding author:

Dr Anwesha Sarkar

Food Colloids and Bioprocessing Group,

School of Food Science and Nutrition, University of Leeds, Leeds LS2 9JT, UK

E-mail address: [A.Sarkar@leeds.ac.uk](mailto:A.Sarkar@leeds.ac.uk)

Supplementary Table S1. Searching terms used across the databases (this example was used in MEDLINE Ovid database 1946-2019) for the current systematic review.

| **Food texture:**  **#1** Food texture.sh. or food texture.ti. or food characteristics.sh. or food characteristics.ti. or food properties.sh. or food properties.ti. or Viscosity.sh. or viscosity.ti. or Semi-solid.sh. or semi-solid.ti. or Liquid.sh. or liquid.ti. or Rheology.sh. or rheology.ti. or Lubrication.sh. or lubrication.ti. |  |
| --- | --- |
| **Appetite:**  **#2** appetite regulation.ti. or appetite control.ti. or appetite.sh. or appetite.ti. or desire to eat.ti. or prospective food consumption.ti. or satiation.sh. or satiation.ti. or hunger.sh. or hunger.ti. or fullness.ti. or thirst.ti. | and |
| **Food intake:**  **#3** Food intake.ti. or energy intake.ti. or food behaviour.ti. or food behaviour.ti. or eating behavio*r | and |
| **Gut hormones:**  **#4** Gut hormones.ti. or gut peptides.ti. or acylated ghrelin.ti. or appetite-related hormones.ti. or appetite-related peptides.ti. or episodic hormones.ti. or episodic peptides.ti. or satiety hormones.ti. or gastrointestinal hormones.sh. or gastrointestinal hormones.ti. or cholecystokinin.ti. or GLP-1.ti. or ghrelin.ti. or PYY.ti. or amylase.ti. or biomarkers.sh. or biomarkers.ti. | and |

Supplementary Table S2. Characteristics of the preloads across the studies included in the systematic review. Measurements units for instrumental and sensorial characteristics are given as in original articles. This table includes also time until next meal (min) and time frame of appetite ratings.

| **Reference** | **Instrumental quantification** | **Sensory quantification** | **Volume/weight** | **Energy density (kcal)** | **Time until next meal (min)** | **Time frame of appetite ratings** |
| --- | --- | --- | --- | --- | --- | --- |
| Camps et al. 2016 | **Viscosity (at 50 s⁻¹ shear rate):**  Thin 100 kcal = 2.9 mPa.s Thin 500 kcal = 11.26 mPa.s Thick 100 kcal = 319 mPa.s Thick 500 kcal = 5897 mPa.s | **Thickness (VAS scale):**  Thin 100 kcal= 14.3±2.6 mm Thin 500 kcal=30.8±4.2 mm Thick 500 kcal=72.7±4.1mm Thick 500 kcal=53.3±4.9 mm | No | Thin/thick = 100 kcal Thin/thick = 500 kcal | 90 | Every 10 min for a total of 90 min |
| Clegg et al. 2012 | No | No | 274 g | No | N/A | Every 15 min for 1 h Every 30 min for 3 h |
| Dong et al. 2016 | **Viscosity (at 65 s⁻¹shear rate):** Liquid = 33±3 cP Semi-solid = 790±12 cP  Solid = 8000±970 cP | No | 240 mL | Liquid = 45 kcal/100 g Semi-solid = 55 kcal/100 g Solid = 36 kcal/100 g | N/A | Every 15 min for 2 h |
| Flood et al. 2007 | **Viscosity (at 10 s⁻¹ shear rate):**  Chunky = 0.7 cps Chunky-pureed = 55 cps Pureed = 235 cps | No | Women = 350 mL  Men = 475 mL | Women = 115 kcal  Men = 156.75 kcal | 15 | On three time points: before, after preload, and after *ad libitum* lunch |
| Flood et al. 2009 | No | No | 266 g | 125 kcal | 15 | On three time points: before, after preload, and after *ad libitum* lunch |
| Hogenkamp et al. 2012 | No | **Thickness (VAS scale)**: LE liquid= 11±15 mm  LE semi-solid = 67±19 mm **Firmness:** LE liquid= 10±18 mm  LE semi-solid= 64±21 mm **Thickness**: HE liquid = 15±20 mm  HE semi-solid = 90±12 mm **Firmness**: HE liquid = 12±19 mm  HE semi-solid = 87±17 mm | Women = 273-330 g  Men = 354-418 g | **Low energy** (liquid and semi-solid) = 30 kcal/100g  **High energy** (liquid and semi-solid) = 130 kcal/100g | Immediately after preload | On three time points: before, after preload, and after *ad libitum* lunch |
| Hogenkamp et al. 2012 | No | **Thickness (VAS scale):** Liquid = 15±14 mm Semi-solid= 85±13 mm | Women = 468 g  Men = 594 g | Liquid = 96 kcal/100g Semi-solid = 98 kcal/100g | N/A | Every 30 min for 3h |
| Juvonen et al. 2011 | **Viscosity (at 50 s⁻¹ shear rate):**  Low viscous = 0.00 Pa s High viscous = 0.32 Pa s **Firmness** **(puncture test):** Low viscous = 0 mN  High viscous = 0 mN  Solid = 5900 mN | No | 400 g | 230 kcal | N/A | Every 15 min for 1h, Hourly for 3h |
| Juvonen et al. 2009 | **Viscosity (at 50s⁻¹ shear rate):** Low viscous = <250 mPas High viscous = >3000 mPas | No | 300 mL | 300 kcal | 180 | Every 15 min for 1h, Hourly for 3h |
| Krop et al. 2019 | **Fracture stress**: Hard/low lubricating= 218 kPa Soft/high lubricating = 27 kPa **Lubrication** **properties (coefficient of friction (µ) at 50 mm s^-1^ speed)**: Hard/low lubricating = 0.26;  Soft/high lubricating = 0.01 | **Chewiness (VAS scale)**: Hard/low lubricating= 77±7mm Soft/high lubricating= 3±2 mm | Women= 25 g Men = 30 g | 0 kcal | Immediately after preload | On three time points: before, after preload, and after *ad libitum* lunch |
| Laboure et al 2002 | No | Unpublished results | **Food 1** = 591 g **Food 2** = 350 g | Type 1= 499 kcal  Type 2= 499 kcal | Approximately after 5-6 h after preload | Every 30 min for 4 h |
| Larsen et al. 2016 | No | **Chewiness rate (chews/s):** Low complex=1.35±4 s High complex=1.40±4s | 32 g | 48 kcal | 10 min | Before, after preload and immediately after lunch and 3 h after lunch |
| Marciani et al. 2012 | **Viscosity (at 50 s⁻¹ shear rate):** of liquid = 509 mPa s | No | 200 g | 240 kcal | N/A | Every 45 min for 3 h |
| Martens et al. 2012 | No | No | Subject specific | Subject specific | N/A | Every 10 min for 1.5 h Every 15 min for another 1.5 h |
| Martens et al. 2011 | No | No | Subject specific | Subject specific | N/A | Every 10 min for 1.5 h Every 15 min for another 1.5 h |
| Mattes 2005 | **Viscosity (at 60 rmp):**  *Food 1* Liquid = 11 cps *Food 2* Liquid = 45 cps **Hardness in terms of solid content (g):** *Food 1* Solid = 770 g *Food 2* Solid=257 g | No | **Food 1** = 652 g **Food 2**: Liquid = 670 g Solid= 199 g | 300 kcal | Food records | Every 15 min for 1.5 h |
| Melnikov et al. 2014 | **Oral stability:**  Aerated = 18% reduction in upon simulated mastication  Non-aerated = 68% reduction in upon simulated mastication | No | **Version 1** Non-aerated = 162 mL Aerated = 500 mL  **Version 2** Non-aerated = 325 mL Aerated = 1,000 mL | Version 1 = 95 kcal Version 2 = 190 kcal | N/A | Every 30 min for 3 h |
| Mourao et al. 2007 | No | No | **Food 1** = 400 g **Food 2:** Liquid = 79 g Solid = 35 g | Food 1 and Food 2 = 125 kcal | Food records | Before and after preload Every hour before leaving the laboratory |
| Santangelo et al. 1998 | **Aperture sieve:** Homogenized = 15% retained on the sieve Solid = 75% retained on the sieve | No | 660 g | 614 kcal | N/A | Every 15 min for 1h, Every 30 min 1.5 h |
|  |  |  |  |  |  |  |
| Solah et al. 2010 | **Viscosity:**  Low viscous = 23.7 cP  High viscous = 27.1 cP | No data | 250 g | 199 kcal | N/A | Before, after preload and every 30 min for 4h |
| Tang et al. 2016 | **Puncture** **(length of curves):**  Low complex = 425.7±4.9 mm High complex = 429.7±35.9 mm | **Chewiness (100 mm scale):** Low complex = 3.13±2.35 mm High complex = 5.24±2.14mm **Hardens:**  Low complex =2.68±2.21mm  High complex =7.10±2.12 mm | 30 g | 40 kcal | 10 min | Before, after preload and immediately after lunch and 3 h after lunch |
| Tournier et al. 1991 | No | No | 538.8 g | 679 kcal | 180 min and food records | Before, after preload and immediately after lunch and 3 h after lunch |
| Tsuchiya et al. 2006 | No | No data shown | 400 mL | 200 kcal | 90 min | Before, after preload and every 10 min for 2 h |
| Wanders et al. 2014 | **Viscosity** **(oral conditions at 100 s⁻¹ shear rate):**  Gels = 1.1 mPa.s  **Viscosity (gastric conditions at 100 s⁻¹ shear rate):** Gels=0.8 mPa.s Capsules=0.8 mPa.s  Liquid = 0.8 mPa.s | No | 425 g | 366 kcal | 180 min | Before, after preload and every 15 min for 3 h |
| Yeomans et al. 2014 | No | **Thickness (VAS scale):** Low sensory= 61.3±3.0 mm High sensory = 68.3±2.6 mm **Creaminess (VAS scale):** Low sensory = 64.2±3.1 mm High sensory = 70.5±2.3 | 320 g | Type 1 = 77 kcal Type 2 = 325 kcal | 90 min | Before and after preload |
| Yeomans et al. 2016 | **Viscosity (at 50 s⁻¹ shear rate):**  Thin = 29.97 mPas  Thick = 333.33 mPas  **Lubrication** **properties (coefficient of friction (µ) at 50 mm s^-1^ speed):** Thin = µ = 0.15 Thick = µ = 0.09 | **Thickness (VAS scale):** *Product 1* Thin = 44.4±3.0 mm Thick = 60.9±2.7 mm  *Product 2* Thin = 48.3±3.0 mm Thick = 64.1±2.8 mm  **Creaminess (VAS scale):** *Product 1* Thin = 53.5±3.3 mm Thick = 60.9±3.2 mm  *Product 2* Thin = 56.1±3.1 mm Thick = 64 ±3.2 mm | 300 mL | Type 1 = 77 kcal Type 2 = 325 kcal | 90 min | Before, after preload, every 30 min for 1.5 h and after lunch |
| Zhu et al. 2013 | **Viscosity (at 30 RPM):**  Standard viscosity = 7,142 cP  High viscosity = 57,142 cP | No | 350 g | 404 kcal | 180 min | Before, after preload, every 15 min for 1.5 h and hourly for 2 h |
| Zhu et al. 2013 | **Viscosity (at 59 RPM shear rate):** Liquid-solid = 10.5 cP  Liquid = 396.7 cP | No | 763 g | 278.7 kcal | 181 min | Before, after preload, every 15 min for 1.5 h and hourly for 2 h |
| Zijlstra et al. 2009 | **Viscosity (at 50 s⁻¹ share rate):**  Liquid = 0.09 Pas  Semi-solid = 2.9 Pas | **Thickness (10 point scale):**  Liquid = 3.4±1.4 cm Semi-solid = 6.0± 0.9 cm | Women = 400 g  Men = 500 g | Women = 388 kcal  Men 485 kcal | 90 min | Before, after and every 30 min for 1.5 h |

Supplementary Table S3. Quality assessment of studies using Cochrane tool.

| Camps 2016 | + | + | - |
| --- | --- | --- | --- |
| Clegg 2013 | + | - | - |
| Dong 2016 | + | + | + |
| Flood 2007 | + | - | - |
| Flood-Obbagy 2009 | + | - | - |
| Hogenkamp 2012 | + | - | - |
| Hogenkamp 2012 | + | - | - |
| Juvonen 2009 | + | - | - |
| Juvonen 2011 | + | - | ? |
| Krop 2019 | + | + | - |
| Laboure 2002 | ? | - | ? |
| Larsen 2016 | + | ? | ? |
| Marciani 2012 | + | ? | ? |
| Martens 2012 | + | ? | - |
| Martens 2011 | + | ? | - |
| Mattes 2005 | ? | ? | - |
| Melnikov 2014 | + | ? | ? |
| Mourao 2007 | - | ? | - |
| Santangelo 1998 | + | ? | ? |
| Solah 2010 | + | ? | - |
| Tang 2016 | + | ? | - |
| Tournier 1991 | - | - | - |
| Tsuchiya 2006 | + | + | ? |
| Wanders 2014 | + | + | - |
| Yeomans 2016 | + | + | ? |
| Yeomans 2014 | + | ? | - |
| Zhu 2013 | + | ? | ? |
| Zhu 2013 | + | ? | ? |
| Zijlstra 2009 | + | + | - |
| \| **+** \| Low risk of bias \| \| --- \| --- \| \| **-** \| High risk of bias \| \| **?** \| Unclear risk of bias \| | Random sequence generation | Allocation concealment | Blinding of participants and personnel |

Supplementary Table S4a. Participants data of studies included in the meta-analysis.

|  |  |  |  |  |  |  |  |  |  |  |  |
| --- | --- | --- | --- | --- | --- | --- | --- | --- | --- | --- | --- |
| **Authors** | **Category** | **C¹** | **I²** | **Male** | **Female** | **Mean Age ± SD** | **Mean BMI ± SD** |  |  |  |  |
| Camps 2016 (low E)⁷ | Viscosity | 15 | 15 | 15 | 0 | 22 ± 2 | 22.6 ± 1.6 |  |  |  |  |
| Camps 2016 (high E)⁷ | Viscosity | 15 | 15 | 15 | 0 | 23 ± 2 | 22.6 ± 1.7 |  |  |  |  |
| Glegg 2013 | Form | 12 | 12 | 6 | 6 | 28.7 ± 5.9 | 23.5 ± 2.9 |  |  |  |  |
| Dong 2016 | Viscosity | 24 | 24 | 17 | 7 | 42 ± 16.16 | 23 ± 2.1 |  |  |  |  |
| Dong 2016 | Form | 24 | 24 | 17 | 7 | 42 ± 16.16 | 23 ± 2.1 |  |  |  |  |
| Flood 2007 | Form | 60 | 60 | 30 | 30 | 26.15 ± 3.87 | 24 ± 2.32 |  |  |  |  |
| Flood 2009 | Form | 58 | 58 | 30 | 28 | 26.95 ± 4.18 | 24 ± 2.88 |  |  |  |  |
| Flood 2009 | Viscosity | 58 | 58 | 30 | 28 | 26.95 ± 4.18 | 24 ± 2.88 |  |  |  |  |
| Hogenkamp 2012 (low E)⁷ | Form | 81³ | 78⁴ | 27 | 54 | 21 ± 2.4 | 22.2 ± 1.6 |  |  |  |  |
| Hogenkamp 2012 (high E)⁷ | Form | 81³ | 81 | 27 | 54 | 21 ± 2.4 | 22.2 ± 1.6 |  |  |  |  |
| Hogenkamp 2012 | Form | 48 | 48 | 9 | 39 | 21 ± 2.9 | 21.8 ± 2 |  |  |  |  |
| Juvonen 2009 | Viscosity | 20 | 20 | 4 | 16 | 22.6 ± 3.13 | 21.6 ± 1.34 |  |  |  |  |
| Juvonen 2011 (Cas)⁷ | Viscosity | 8 | 8 | 8 | 0 | 24 ± 2.31 | 23.3 ± 1.41 |  |  |  |  |
| Juvonen 2011 (TG-Cas)⁷ | Viscosity | 8 | 8 | 8 | 0 | 24 ± 2.31 | 23.3 ± 1.41 |  |  |  |  |
| Juvonen 2011 (WP)⁷ | Viscosity | 8 | 8 | 8 | 0 | 24 ± 2.31 | 23.3 ± 1.41 |  |  |  |  |
| Laboure 2002 (veg.)⁷ | Form | 12 | 12 | 12 | 0 | 21.5 ± 2.07 | 22.28 ± 1.93 |  |  |  |  |
| Laboure 2002 (rsuk) | Form | 12 | 12 | 12 | 0 | 21.5 ± 2.07 | 22.28 ± 1.93 |  |  |  |  |
| Marciani 2012 | Form | 22 | 22 | 13 | 9 | 29 ± 4.22 | 21.1 ± 3.75 |  |  |  |  |
| Martens 2011 | Form | 10 | 10 | 10 | 0 | 21.1 ± 3.9 | 22.4 ± 1.2 |  |  |  |  |
| Martens 2012 | Form | 10 | 10 | 10 | 0 | 21.1 ± 4.11 | 22.4 ± 1.26 |  |  |  |  |
| Mattes 2005 (CHO)⁷ | Form | 31 | 31 | 13 | 18 | 23.7 ± 5 | 23 ± 3.9 |  |  |  |  |
| Mattes 2005 (protein) | Form | 31 | 31 | 13 | 18 | 23.7 ± 5 | 23 ± 3.9 |  |  |  |  |
| Solah 2010 (ALG)⁷ | Viscosity | 33 | 33 | 16 | 17 | 21.2 ± 1.8 | 22.7 ± 1.81 |  |  |  |  |
| Solah 2010 (WP)⁷ | Viscosity | 33 | 33 | 16 | 17 | 21.2 ± 1.8 | 22.7 ± 1.81 |  |  |  |  |
| Tournier 1991 | Form | 13 | 13 | 7 | 6 |  |  |  |  |  |  |
| Tsuchiya 2006 | Form | 32 | 32 | 16 | 16 | 27.1 ± 4.7 | 22.9 ± 1.9 |  |  |  |  |
| Wanders 2014 (capsule) | Form | 29 | 29 | 29 | 0 | 21 ± 2 | 21.9 ± 2.8 |  |  |  |  |
| Wanders 2014 (gel) | Form | 29 | 29 | 29 | 0 | 21 ± 2 | 21.9 ± 2.8 |  |  |  |  |
| Yeomans 2014 (low E)⁷ | Viscosity | 12⁵ | 12 | 12 | 0 | 20.4 ±1.73 | 23.65 ± 2.77 |  |  |  |  |
| Yeomans 2014 (high E)⁷ | Viscosity | 12⁵ | 12 | 12 | 0 | 22.2 ±1.38 | 24.1 ± 3.46 |  |  |  |  |
| Yeomans 2016 (low E)⁷ | Viscosity | 22 | 22 | 22 | 0 | 31⁶ | 24 |  |  |  |  |
| Yeomans 2016 (high E)⁷ | Viscosity | 22 | 22 | 22 | 0 | 31⁶ | 24 |  |  |  |  |
| Zhu 2013 | Viscosity | 15 | 15 | 15 | 0 | 27 ± 2 | 24.2 ± 2.32 |  |  |  |  |
| Zhu 2013 | Form | 19 | 19 | 19 | 0 | 28 ± 2 | 24.2 ± 2.61 |  |  |  |  |
| Zijlstra 2009 | Form | 32 | 32 | 12 | 20 | 22 ± 2 | 21.9 ± 2.2 |  |  |  |  |
|  |  |  |  |  |  |  |  |  |  |  |  |
|  |  |  |  |  |  |  |  |  |  |  |  |
| ¹ Control/comparison |  |  |  |  |  |  |  |  |  |  |  |
| ² Intervention |  |  |  |  |  |  |  |  |  |  |  |
| ³ Participants had the preload 3 times a day, 27*3=81 | | |  |  |  |  |  |  |  |  |  |
| ⁴ Data on 2 participants missing |  |  |  |  |  |  |  |  |  |  |  |
| ⁵ Between-participants study design | |  |  |  |  |  |  |  |  |  |  |
| ⁶ Data missing on standard deviation | |  |  |  |  |  |  |  |  |  |  |
| ⁷ **Abbreviations**:  **low E** = low energy  **high E**= high energy  **Cas** = casein  **TG-Cas** = transglutaminate treated casein  **WP** = whey protein  **veg**. = vegetables  **CHO** = carbohydrates  **ALG** = alginate |  |  |  |  |  |  |  |  |  |  |  |

Supplementary Table S4b. Meta-analysis data on appetite ratings (hunger and fullness).

|  |  | | |  | |  |  |  |  |  |  |  |  |  |
| --- | --- | --- | --- | --- | --- | --- | --- | --- | --- | --- | --- | --- | --- | --- |
| **Authors** | **Category** | | | **C¹** | | **I²** | **C¹ Mean Hunger** | **C¹ SD Hunger** | **I² Mean Hunger** | **I² SD Hunger** | **C¹ Mean Fullness** | **C¹ SD Fullness** | **I² Mean Fullness** | **I² SD Fullness** |
| Camps 2016 (low E) | Viscosity | | | 15 | | 15 |  |  |  |  | 38.75 | 2.75 | 45 | 1.37 |
| Camps 2016 (high E) | Viscosity | | | 15 | | 15 |  |  |  |  | 42.5 | 1.37 | 48.75 | 2.75 |
| Glegg 2013 | Form | | | 12 | | 12 |  |  |  |  | 57 | 13.32 | 40 | 21.64 |
| Dong 2016 | Viscosity | | | 24 | | 24 | 43.75 | 17.63 | 37.5 | 19.59 | 28.75 | 19.59 | 40 | 19.59 |
| Dong 2016 | Form | | | 24 | | 24 | 43.75 | 17.63 | 36.25 | 19.59 | 28.75 | 19.59 | 37.5 | 19.59 |
| Flood 2007 | Form | | | 60 | | 60 | 31.25 | 24.16 | 35.4 | 24.16 |  |  |  |  |
| Hogenkamp 2012 (low E) | Form | | | 81³ | | 78⁴ | 57.3 | 17.3 | 46 | 19.4 |  |  |  |  |
| Hogenkamp 2012 (high E) | Form | | | 81 | | 81 | 63.3 | 16.4 | 55.2 | 16.7 |  |  |  |  |
| Hogenkamp 2012 | Form | | | 48 | | 48 | 29.07 | 11.43 | 18.18 | 11.43 | 78.18 | 11.43 | 63.63 | 19.05 |
| Juvonen 2011 (WP) | Viscosity | | | 8 | | 8 | 32.5 | 28.28 | 32.5 | 14.14 | 37.5 | 22.62 | 37.5 | 16.97 |
| Juvonen 2011 (Cas) | Viscosity | | | 8 | | 8 | 32.5 | 28.28 | 25 | 11.31 | 37.5 | 22.62 | 53.75 | 33.94 |
| Juvonen 2011 (TG-Cas) | Viscosity | | | 8 | | 8 | 32.5 | 14.14 | 25 | 11.31 | 37.5 | 16.97 | 53.75 | 33.94 |
| Laboure 2002 (veg.) | Form | | | 12 | | 12 | 20 | 14.97 | 20 | 11.84 | 71.4 | 11.84 | 65.7 | 24.66 |
| Laboure 2002 (rusk) | Form | | | 12 | | 12 | 32.82 | 24.66 | 27.12 | 15.27 | 51.4 | 19.74 | 62.85 | 12.81 |
| Marciani 2012 | Form | | | 22 | | 22 |  |  |  |  | 3.71 |  | 3.35 |  |
| Martens 2011 | Form | | | 10 | | 10 | 32.5 | 18.97 | 20 | 12.64 | 65 | 31.62 | 77.5 | 15.81 |
| Martens 2012 | Form | | | 10 | | 10 |  |  |  |  | 76.66 | 10.65 | 70.99 | 21.06 |
| Mattes 2005 (CHO) | Form | | | 31 | | 31 | 36.53 | 12.69 | 25.7 | 22.21 | 68.55 | 3.17 | 80 | 6.34 |
| Mattes 2005 (protein) | Form | | | 31 | | 31 | 36.53 | 12.69 | 40 | 31.73 | 68.55 | 3.17 | 65.7 | 3.17 |
| Solah 2010 (ALG) | Viscosity | | | 33 | | 33 | 16.65 | 9.53 | 16.65 | 9.53 |  |  |  |  |
| Solah 2010 (WP) | Viscosity | | | 33 | | 33 | 11.65 | 9.53 | 16.65 | 9.53 |  |  |  |  |
| Tournier 1991 | Form | | | 13 | | 13 | 20 | 18.02 | 10 | 8 |  |  |  |  |
| Wanders 2014 (capsule) | Form | | | 29 | | 29 | 34.98 | 17.93 | 28.32 | 16.1 |  |  |  |  |
| Wanders 2014 (gel) | Form | | | 29 | | 29 | 34.98 | 17.93 | 34.98 | 17.93 |  |  |  |  |
| Yeomans 2014 (low E) | Viscosity | | | 12⁴ | | 12 | -8 |  | -21 |  | 14 |  | 28 |  |
| Yeomans 2014 (high E) | Viscosity | | | 12⁴ | | 12 | -16 |  | -28 |  | 18 |  | 26 |  |
| Yeomans 2016 (low E) | Viscosity | | | 19 | | 19 | 2.13 |  | 0.71 |  | 1.77 |  | 1.42 |  |
| Yeomans 2016 (high E) | Viscosity | | | 19 | | 19 | -2.13 |  | -6.06 |  | 0 |  | -13.62 |  |
| Zhu et al. 2013 | Viscosity | | | 15 | | 15 | 30 | 15.49 | 26 | 3.87 | 50 | 5.8 | 60 | 7.35 |
| Zhu et al. 2013 | Form | | | 19 | | 19 | 26.49 |  | 23.51 |  | 30 |  | 26.85 |  |
| Zijlstra et al. 2009 | Form | | | 32 | | 32 | 52.5 | 15 | 52.5 | 17.5 | 51 | 11.25 | 50 | 16.25 |
|  |  | | |  | |  |  |  |  |  |  |  |  |  |
|  |  | | |  | |  |  |  |  |  |  |  |  |  |
| ¹ Control/comparison | |  |  | |  |  |  |  |  |  |  |  |  |  |
| ² Intervention | |  |  | |  |  |  |  |  |  |  |  |  |  |
| ³ Participants had the preload 3 times a day, 27*3=81 | | | | |  |  |  |  |  |  |  |  |  |  |
| ⁴ Data on 2 participants missing | |  |  | |  |  |  |  |  |  |  |  |  |  |
| ⁵ Between-participants study design | | |  | |  |  |  |  |  |  |  |  |  |  |

Supplementary Table S4c. Meta-analysis data on food intake.

|  |  |  |  |  |  |  |  |
| --- | --- | --- | --- | --- | --- | --- | --- |
| **Authors** | **Category** | **C¹** | **I²** | **C¹ Food intake Mean** | **C¹ Food intake SD** | **I² Food intake Mean** | **I² Food intake SD** |
| Camps 2016 (low E) | Viscosity | 15 | 15 | 625.71 | 365.43 | 476.81 | 343.69 |
| Camps (high E) | Viscosity | 15 | 15 | 541.58 | 283.46 | 531.07 | 321.46 |
| Flood 2007 | Form | 60 | 60 | 654 | 340 | 704 | 371 |
| Flood 2009 | Form | 58 | 58 | 800 | 373 | 709 | 380 |
| Flood 2009 | Viscosity | 58 | 58 | 890 | 388 | 866 | 396 |
| Hogenkamp 2012 (low E) | Form | 81³ | 78⁴ | 1767 | 581 | 1720 | 583 |
| Hogenkamp 2012 (high E) | Form | 81 | 81 | 1549 | 427 | 1496 | 438 |
| Juvonen 2009 | Viscosity | 20 | 20 | 2007 | 154 | 1733 | 113 |
| Laboure 2002 (veg.) | Form | 12 | 12 | 777 | 301 | 791 | 204 |
| Laboure 2002 (rusk) | Form | 12 | 12 | 940 | 301 | 704 | 377 |
| Tournier 1991 | Form | 13 | 13 | 781 | 259.29 | 769 | 407.42 |
| Tsuchiya 2006 | Form | 32 | 32 | 803 | 299.81 | 776 | 282.84 |
| Wanders 2014 (capsule) | Form | 29 | 29 | 1128.84 | 332.51 | 1058 | 339.24 |
| Wanders 2014 (gel) | Form | 29 | 29 | 1128.84 | 332.51 | 955.61 | 305.74 |
| Yeomans 2014 (low E) | Viscosity | 12⁵ | 12 | 942.74 | 266.77 | 1022.41 | 193.15 |
| Yeomans 2014 (high E) | Viscosity | 12⁵ | 12 | 873.69 | 275.95 | 677.33 | 193.15 |
| Yeomans 2016 (low E) | Viscosity | 22 | 22 | 1337.5 | 75 | 1375 | 87.5 |
| Yeomans 2016 (high E) | Viscosity | 22 | 22 | 1282.5 | 75 | 1200 | 80 |
| Zhu 2013 | Viscosity | 15 | 15 | 791.2 | 80 | 788.8 | 82.88 |
| Zhu 2013 | Form | 19 | 15 | 878.96 | 80.32 | 882.4 | 88 |
| Zijlstra 2009 | Form | 32 | 32 | 394.92 | 212.94 | 371.69 | 178.1 |
|  |  |  |  |  |  |  |  |
|  |  |  |  |  |  |  |  |
| ¹ Control/comparison |  |  |  |  |  |  |  |
| ² Intervention |  |  |  |  |  |  |  |
| ³ Participants had the preload 3 times a day, 27*3=81 | | |  |  |  |  |  |
| ⁴ Data on 2 participants missing |  |  |  |  |  |  |  |
| ⁵ Between-participants study design | |  |  |  |  |  |  |
|  |  |  |  |  |  |  |  |

Hunger

a)

Fullness

b)

Food intake

c)

Supplementary Figure S1. Funnel plots of food texture effects on hunger (a), fullness (b) and food intake (c) with a 95% confidential interval (CI). The data (blue dots) indicate each study included in meta-analysis. The more symmetric the plots are, the less is the publication bias of the studies.
